# Size Matters in Conjugated Polymer Chirality‐Selective SWCNT Extraction

**DOI:** 10.1002/advs.202402176

**Published:** 2024-05-24

**Authors:** Andrzej Dzienia, Dominik Just, Tomasz Wasiak, Karolina Z. Milowska, Anna Mielańczyk, Norman Labedzki, Sebastian Kruss, Dawid Janas

**Affiliations:** ^1^ Department of Chemistry Silesian University of Technology B. Krzywoustego 4 Gliwice 44–100 Poland; ^2^ CIC Nanogune Donostia‐San Sebastián 20018 Spain; ^3^ Ikerbasque Basque Foundation for Science Bilbao 48013 Spain; ^4^ Department of Chemistry Ruhr‐University Bochum 44801 Bochum Germany; ^5^ Biomedical Nanosensors Fraunhofer Institute for Microelectronic Circuits and Systems 47057 Duisburg Germany

**Keywords:** conjugated polymers, purification, single‐walled carbon nanotubes, sorting

## Abstract

Carbon‐based nanomaterials have catalyzed breakthroughs across various scientific and engineering disciplines. The key to unlocking a new generation of tailor‐made nanomaterials based on single‐walled carbon nanotubes (SWCNTs) lies in the precise sorting of raw material into individual chiralities, each possessing unique properties. This can be achieved using conjugated polymer extraction (CPE), but to a very limited extent since the process generates only a few chirality‐enriched suspensions. Therefore, it is imperative to comprehend the mechanism of the wrapping of SWCNTs by polymers to unleash CPE's full potential. However, the lack of a diverse palette of chirality‐selective polymers with varying macromolecular parameters has hindered a comprehensive understanding of how the nature of the polymer affects the performance and selectivity of SWCNT isolation. To address this gap, multiple batches of such polymers are synthesized to elucidate the impact of molecular weight and dispersity on the purity and concentrations of the generated SWCNT suspensions. The obtained results explain the inconsistent outcomes reported in the literature, greatly improving the application potential of this promising SWCNT sorting approach. Concomitantly, the discovered significant influence of the macromolecular characteristics of conjugated polymers on the SWCNT isolation efficacy sheds considerable insight into the unresolved mechanism of this sorting technique.

## Graphical abstract

This work reveals how the molecular characteristics of conjugated polymers make a substantial impact on the course of the conjugated polymer extraction of single‐walled carbon nanotubes. By adjusting the size of the polymer molecules present in the system and gradually increasing their concentration in the liquid medium, the selectivity, and yield of the extraction of single‐walled carbon nanotubes can be dramatically enhanced.

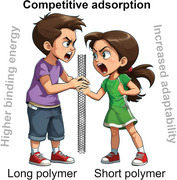

## 1 Introduction

Sweeping progress has been made^[^ ^1^, ^2^, ^3^, ^4^ ^]^ since conjugated polymer extraction (CPE) that enabled selective isolation of single‐walled carbon nanotubes (SWCNTs) in organic solvents was discovered.^[^ ^5^ ^]^ To date, scientists have discovered several conjugated polymers (CPs) that exhibit a preference for certain SWCNT chiralities. From the point of view of widespread applicability, it is worth distinguishing three of them that enable near‐monochiral resolution: poly(9,9‐dioctylfluorene‐2,7‐diyl‐*alt*‐6,6′‐{2,2′‐bipyridine}) (PFO‐BPy6,6′), poly(9,9‐dioctylfluorene‐2,7‐diyl) (PFO), and poly(9,9‐dioctylfluorene‐2,7‐diyl)‐*alt*‐(benzothiadiazole) (PFO‐BT/F8BT), with remarkable affinity to (6,5),^[^ ^6^, ^7^ ^]^ (7,5),^[^ ^5^, ^8^ ^]^ and (7,3) SWCNTs,^[^ ^9^ ^]^ respectively.

Through numerous studies in this area, scientists have gained valuable, although fragmentary and still somewhat limited, knowledge about the influence of 1) the composition of the extraction system,^[^ ^7^ ^]^ 2) solvent selection,^[^ ^9^, ^10^, ^11^, ^12^, ^13^, ^14^, ^15^ ^]^ 3) temperature, 4) the presence of the polymer β‐phase,^[^ ^15^, ^16^ ^]^ and 5) mixture viscosity^[^ ^12^ ^]^ on the selectivity and efficiency of SWCNT extraction. Unfortunately, understanding the mechanism of chirality‐selective isolation itself, or at least identifying its thermodynamic or kinetic basis, has not been fully successful.^[^ ^3^ ^]^ Many of the abovementioned factors have been analyzed while differentiating semiconducting (s‐SWCNTs) and metallic SWCNTs (m‐SWCNTs), which is much less restrictive than sorting SWCNTs by chirality. In the former case, selective isolation of s‐SWCNTs versus m‐SWCNTs can usually be achieved for many structurally distinct polymers, using both polar and non‐polar solvents, by applying a cross‐section of polymer to SWCNT weight ratios, or even different SWCNT source materials.^[^ ^17^, ^18^ ^]^ Since m‐SWCNTs are several orders of magnitude more polarizable than s‐SWCNTs, they are effectively precipitated by CPE, providing a highly‐enriched suspension of s‐SWCNTs, which are of particular value for microelectronics due to the presence of bandgap.^[^ ^19^ ^]^ Chirality sorting, on the other hand, usually requires a meticulous selection of parameters to achieve the optimal process mixture composition (type and relative amount of SWCNTs, CPs, and solvent) and purification conditions (sonication power, centrifugation force, etc.).^[^ ^8^, ^15^, ^20^, ^21^, ^22^ ^]^ A sub‐optimal combination of parameters, such as the composition of raw SWCNTs or the macromolecular characteristics of the polymer, can very easily reduce the selectivity of the process or even make the process chirality indiscriminate.

For metallic/semiconducting SWCNT sorting, it is essential to find a sufficiently flexible conjugated polymer capable of forming π‐bonds with the SWCNT sidewall. Provided that the polymer offers an appropriate bandgap and electron density, charge transfer promoting such differentiation may occur.^[^ ^17^, ^18^, ^23^ ^]^ However, for chirality selective isolation, the spatial conformation of the polymer is of much greater relevance. The polymer conformation should be suitable for a small fraction of chiral diameters and angles, ideally preferring only a single SWCNT type present in the processed raw material.

Beyond doubt, the macromolecular parameters of the polymer are one of the most crucial factors determining the application potential of such material in this context. There is a direct correlation between the length of the polymer chain and properties such as phase transition temperatures, intrinsic viscosity, and the potential for the formation of local spatial ordering known as the β‐phase.^[^ ^24^ ^]^ The latter has an indisputable, although scarcely validated, effect on selectivity and isolation efficiency.^[^ ^15^, ^16^ ^]^ An additional source of system complexity is the interrelation of the abovementioned aspects with the choice of solvent in which the isolation process is carried out.^[^ ^12^, ^15^ ^]^ Regrettably, despite having such an essential role in the CPE process, the influence of polymer molecular weight on the mechanism of the SWCNT wrapping process and, thus, the yield and selectivity of the chirality‐selective sorting have not been adequately addressed. Thus, even for the most well‐known selective isolation systems, e.g., SWCNT/PFO‐BPy6,6′ or SWCNT/PFO, the relationships between the macromolecular parameters of the polymer and the optimal isolation procedure/process mixture composition are unknown. The major challenge that hinders such long‐desired studies is the necessity to reach appropriate control over the polymerization process to obtain polymers with the desired molecular weights necessary to carry out such investigations. This effect is especially challenging in the case of alternating conjugated copolymers, for which controlled polymerization methods are out of reach or, at best, very limited.^[^ ^25^, ^26^ ^]^ Consequently, the polymers useful for CPE are scarcely available on the market and often, materials of incompatible macromolecular properties and dispersity indices are offered. This issue could be circumvented by the fractionation of the conjugated polymers. However, due to the requirement for specialized equipment, i.e., preparative GPC/SEC with appropriate columns combined with the limited solubility of such CPs, this strategy is rarely implemented.^[^ ^24^ ^]^ Similarly, scant attention has been paid by the scientific community to the optimal weight ratio of polymer to the raw polychiral SWCNT mixture, this aspect being commonly neglected in the literature. Hence, a wide range of CP:SWCNT weight ratios, from 0.5:1 to 50:1,^[^ ^27^ ^]^ have been used, without providing any reason for such a drastic variation in extraction conditions.

In this study, we synthesized conjugated polymers through Suzuki polycondensation, while attempting to adjust their macromolecular properties by modifying the composition of the reaction mixture, manipulating process time and temperature, or activating the catalytic system using temperature and microwave radiation.^[^ ^28^ ^]^ The integration of microwave radiation with recently developed catalytic systems, featuring nickel nanowires decorated with palladium nanoparticles, has demonstrated profound advantages in the field of chemical synthesis.^[^ ^29^ ^]^ This synergistic approach has significantly increased the range of achievable molecular weights while leading to a substantial reduction in reaction times by orders of magnitude. The list of polymers obtained, along with a description of the employed synthesis conditions, is depicted in Table S1 (Supporting Information). Based on the library of conjugated polymers (PFO and PFO‐BPy6,6′) obtained this way, we unraveled the previously unclear effect of polymer molecular weight on the selectivity and yield of model CPE‐based SWCNT purification systems. Finally, the relationship between polymer size and optimal CP:SWCNTs ratio was analyzed for a few selected, representative conjugated polymers. Consequently, we established optimal conditions for harvesting chirality‐defined SWCNTs on a large scale, which should immensely enhance the application potential of this promising material. Importantly, the discovered polymer‐nanomaterial‐solvent interactions, due to their interdisciplinary importance, may facilitate the understanding of other phenomena in various sectors of nanoscience.

## 2 Results and Discussion

**Figure 1:**
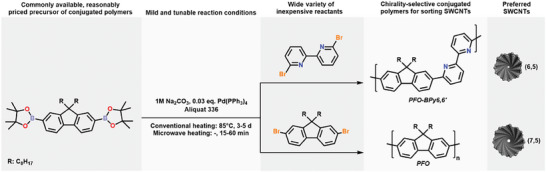
General Suzuki Polycondensation (SPC) scheme.

To investigate the effect of the molecular weight of the conjugated polymers on the performance of the chirality‐selective isolation via CPE process, we selected two model polymers already mentioned in the introduction, i.e., polydioctylfluorene (PFO) and polydioctylfluorene‐*alt*‐6,6‐bipyridyl (PFO‐BPy6,6′) (**Figure** **1**), which favor the isolation of (7,5) and (6,5) SWCNTs, respectively. In our previous studies, we noted that these polymers exhibit differences in behavior with regard to their macromolecular properties, the composition of raw SWCNTs, and that of the process mixture, but at that time, we did not have a sufficiently broad array of polymer batches to investigate these phenomena in detail.^[^ ^9^, ^30^ ^]^ Synthesis of a wide spectrum of polymers of different characteristics, employing the Suzuki coupling approach (procedures explained in the Supporting Information), as well as the conducted computational work, enabled us to interpret these dissimilarities for the first time herein.

**Figure 2:**
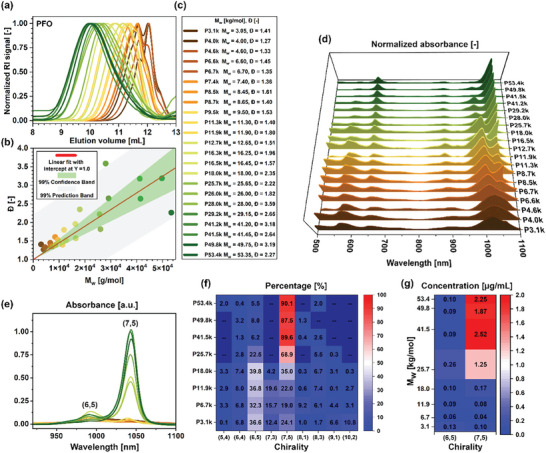
a) GPC chromatograms of the obtained PFOs. b) Correlation between weight‐average molecular weight (M_w_) and dispersity. c) Abbreviations used in this study denote specific PFO batches. d) Optical absorbance spectra normalized to the highest intensity peaks displaying S_11_ transitions of specific SWCNTs and e) as received optical absorbance spectra enabling, respectively, qualitative and quantitative evaluation of the suitability of particular PFOs for isolation of (7,5) SWCNTs. f) Percentage values and g) concentrations of various SWCNTs from deconvolution of selected absorbance spectra given in panel (e). The spectra were obtained under model CPE process conditions (1.5 mg SWCNTs + 9 mg PFO + 4.5 mL toluene).

PFO was the first polymer to be investigated. Due to its high accessibility stemming from its use in LEDs,^[^ ^31^ ^]^ it has become one of the most widely used representatives of fluorene‐based conjugated polymers, often employed to study selective extraction of SWCNTs via CPE.^[^ ^5^, ^8^, ^15^ ^]^ Even so, the selection of a polymer with the desired characteristics when procuring materials for SWCNT purification is usually impossible because vendors typically do not report these parameters for a given batch. **Figure** **2**a shows superimposed GPC chromatograms of a whole series of synthesized and examined PFO batches together with the exact characteristics shown in Figure 2b. Looking at the distribution of the dispersity parameter with respect to the weight‐average molecular weight of the polymers, M_w_ (Figure 2c), we noted that it fluctuated around the linear relationship determined in Figure 2b, meaning that, in the case of PFO, the synthesis was characterized by a moderate control typical for a polycondensation mechanism.

Based on such a vast and diverse cross‐section of PFO batches with different macromolecular parameters, we proceeded to study their utility for conducting the CPE process. A detailed characterization of the SWCNT crude material, along with the determination of the content of particular chiralities, was described in our previous publication.^[^ ^9^, ^32^ ^]^ In brief, the raw SWCNTs selected for this study (CoMoCAT SWCNTs enriched with (6,5) chirality) were dominated by several major types. The first one (occurring in large excess) was (6,5), whose estimated percentage content was ≈40–50%, followed by (7,5) and (7,3) with ≈10–15% content each. Besides them, slightly less abundant were (6,4) and (8,3), estimated at 7–10%, (8,4), and (7,6) at ≈5%, and (9,1), (8,1), (5,4), and (9,2) present in negligible amounts.

The supernatants obtained from the extraction of SWCNTs using these polymers were analyzed by means of optical absorption spectroscopy (Figure 2d,e). To quantify the acquired experimental data, the selected spectra were deconvoluted using the PTF Fit program (according to the procedure described in Supplementary Information, examples of data processing are depicted in Figure S5, Supporting Information)^[^ ^33^ ^]^ to determine the percentage and concentration of each chirality (Figure 2f,g). The final section of our analysis involved using the calculated concentrations to calculate an enrichment factor (EF), which took into account how the concentrations of individual chiralities in the supernatant referred to their initial concentration in the raw material (methodology explained in Figure S6, Supporting Information).

The first few polymers with the smallest M_w_, i.e., below 11.9 kg mol^−1^, suspended a minor fraction of the SWCNTs available in the raw material without any apparent preference (Figure 2d). This was revealed by the fact that the spectra had a high level of background, and the peaks from individual chiralities were not clearly marked. Most likely, it was the result of the solubilization of small SWCNT clusters consisting of different chiralities rather than forming well‐individualized and selectively wrapped SWCNTs. The low intensity of the recorded spectra confirmed that small amounts of SWCNTs were suspended with these polymers (Figure 2e,g). Moreover, these materials provided statistical solubilization of SWCNTs, as the results resembled the spectra of (6,5)‐enriched CoMoCAT SWCNTs suspended non‐selectively with surfactants in water.^[^ ^34^ ^]^ The next polymers, with M_w_ above 11.3 up to 12.7 kg mol^−1^ (e.g., sample P12.7k), displayed slightly improved individualization of the (6,5) and (7,5) chiralities, as the corresponding peaks were better defined. Still, the polymer did not show a satisfactory level of enrichment, most likely because the amount of (7,5) SWCNTs in the raw material was three times lower than (6,5), and the examined polymers of such molecular weight range did not exhibit sufficiently high selectivity. Here, it is worth highlighting that no conjugated polymer exhibits an exclusive interaction with only a single chirality. This means that replacing the starting material with one containing a distinct diameter or chiral angle distribution results in an adaptation of polymer selectivity toward the most preferred species.^[^ ^7^, ^15^ ^]^

For M_w_ in the range of 16.6–18.0 kg mol^−1^, the (7,5) chirality began to prevail over (6,5)‐SWCNTs, although the latter was still discernible (Figure 2f,g). A monochiral selectivity of isolation using our model procedure was finally achieved with polymers of M_w_≈30 kg mol^−1^ (corresponding to M_n_ ≈10–12 kg mol^−1^, with an average Đ ≈2.5–3.0). The best of the investigated polymers gave a (7,5) purity of ≈90% (Figure 2f) with respect to all identified chiralities. Only (6,5) SWCNTs, the most abundant species in the parent material, could be identified as an impurity with certainty in the registered optical absorbance spectra (Figure 2d,e). However, the intensity of the peak corresponding to such SWCNTs was low. In addition, subsequent characterization conducted using photoluminescence excitation‐emission mapping revealed a negligible presence of such SWCNTs in selected samples with high estimated purity (except for the P25.7k SWCNT suspension, which was produced using too short PFO polymer).

Lastly, the concentration of the produced dispersions was notably high, exceeding 2.0 µg mL^−1^ for SWCNTs sorted using two of the high molecular weight PFO samples, namely P41.5 and P53.4k (Figure 2g). Therefore, not only did the selectivity evolve significantly with the increase in the molecular weight of the polymer, but the effectiveness of SWCNT suspension increased as well. Batches containing a high share of fractions below 12 kg mol^−1^ were characterized by a mediocre ability to solubilize SWCNTs, which translated into absorbance levels below 0.1 a.u. On the other hand, when the weight distribution shifted toward higher MW fractions and, at the same time, the dispersity increased proportionally, the extraction yield started to increase rapidly, without any negative impact on the selectivity. For instance, P25.7k (M_w_ = 25.65, Đ = 2.22) and P28.0k (M_w_ = 28.0, Đ = 3.59) polymers illustrate this behavior well, both of which produced SWCNT suspensions reaching an absorbance of ca. 0.5 a.u (Figure 2e).

By analyzing the enrichment factor, as depicted in Figure S6a (Supporting Information), we elucidated the variations in chirality preference for (6,5) and (7,5) with the increasing chain length of PFO. We observed that even for short polymer chains, i.e., molecular weights below M_w_ ≈11.9 kg mol^−1^, PFO had a slightly higher affinity for (7,5) than for (6,5), as manifested by an EF of 1.05–1.43 versus 0.65–0.72, respectively. Values below 1 indicated that the amount of dispersed (6,5) was reduced compared to its content in the crude material. The increase in EF toward (7,5) started for M_w_ ≥11.9 kg mol^−1^ and reached its maximum (EF≈4.2–4.3) at molecular weights ≈41 kg mol^−1^. In the case of (6,5), the decrease in EF values occurred for polymers with M_w_ ≈18 kg mol^−1^, and the lowest EFs were also observed for PFOs heavier than 41 kg mol^−1^, reaching values as low as 0.09.

**Figure 3:**
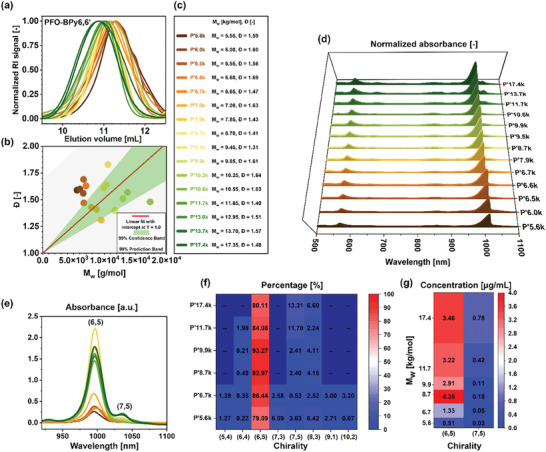
a) GPC chromatograms of the obtained PFO‐BPy6,6′ polymers. b) Correlation between weight‐average molecular weight (M_w_) and dispersity. c) Abbreviations used in this study denoting specific PFO‐BPy6,6′ batches. d) Optical absorbance spectra normalized to the highest intensity peaks displaying S_11_ transitions of specific SWCNTs and e) as received optical absorbance spectra enabling, respectively, qualitative and quantitative evaluation of the suitability of particular PFO‐BPy6,6′ for isolation of (6,5) SWCNTs. f) Percentage values and g) concentrations of various SWCNTs from deconvolution of selected absorbance spectra given in panel (d). The spectra were obtained under model CPE process conditions (1.5 mg SWCNTs + 6 mg PFO‐BPy6,6′ + 4.5 mL toluene).

To check whether the above‐discussed findings were universal and described the interactions of SWCNTs with CPs of any type, we used another polymer (PFO‐BPy6,6′), and the results of this investigation are presented in **Figure** **3**. It is the most widely implemented and investigated example of a chirality‐selective CP, exhibiting unparalleled affinity toward (6,5) SWCNTs.^[^ ^6^ ^]^ Interestingly, in contrast to the previously discussed PFO, whose various synthesis methods have been described in numerous scientific papers, PFO‐BPy6,6′, regardless of the nanocarbon research group under consideration, came almost exclusively from the same vendor.^[^ ^35^, ^36^, ^37^, ^38^ ^]^ To address the question of whether this is related to the challenging synthetic pathway or rather the lack of literature examples, we performed a series of polymerization reactions utilizing analogous conditions to those used for PFO in both conventional heating and microwave reactor procedures (according to the procedure described in Supporting Information).

An adjustment in the synthetic protocol by increasing the polymerization time for conventional heating to 5–6 days was necessary due to the lower reactivity of this reaction system. Further extending the reaction time no longer resulted in a clear increase in yield and molecular weight of the produced polymers. Notwithstanding these optimization endeavors, most of the obtained polymers had a molecular weight that did not exceed 20 kg mol^−1^ (Figure 3a–c). Only when the synthesis was scaled up, high molecular‐weight batches of PFO‐BPy6,6' were obtained. The obtained results strongly suggested that the limited likelihood of obtaining polymers with higher molecular weights, might be related to thermodynamic or kinetic limitations.

These issues are plausibly linked with the up to several times lower solubility of PFO‐BPy6,6′ compared to PFO (even considering that typical PFOs have a much higher M_w_), which we experienced while working with these polymers (estimated values of solubility for polymers with different molecular weights and some handling suggestions are included in the Supporting Information). Thus, it is likely that spontaneous precipitation of polymers with higher chain lengths occurred during the synthesis, preventing further efficient elongation. Additionally, solubility issues may lead to the possible depletion of certain fractions during the purification process. Additionally, the inferior reactivity of the pyridyl group, regarded as one of the most difficult moieties in cross‐coupling reactions,^[^ ^39^ ^]^ hindered the opportunity for the synthesis of PFO‐BPy6,6′ with large molecular weights. Undoubtedly, this issue requires further research from the polymer chemistry point of view, especially if the importance of the chirality‐selective isolation of SWCNTs continues to expand.

Regardless of the challenges in producing PFO‐BPy6,6′ with an M_w_ higher than 12 kg mol^−1^, the results of SWCNT extraction demonstrated that sufficiently high selectivity and yield could be obtained at lower M_w_ values (Figure 3d–g). PFO‐BPy6,6′ exhibited remarkable selectivity toward (6,5)‐chirality starting from the lowest molecular weight examined, i.e., 5.6 kg mol^−1^, yielding an absorbance value of 0.3 a.u., which was already competitive with respect to the literature data. Upon increasing the molecular weight by only 1 kg mol, the yield increased to 0.6 a.u. Interestingly, continued elongation of the polymer enabled very concentrated SWCNT suspensions (1.4–2 a.u.) to be reached for polymers with M_w_ of ≈10 kg mol^−1^ and dispersity Đ≈1.3–1.4 (Figure 3b). Such high absorbance values also translated into very high concentrations of 2.9 to as much as 4.4 µg mL^−1^ (Figure 3g). Most importantly, we noted that the increased yield was not reached at the expense of purity, which in this case was as high as 93%, meeting the criteria for monochirality. Also, polymers outside the optimal molecular weight range offered highly selective isolation, resulting in a generally acceptable purity of ≈80–85%. The further increase in the molecular weight of the polymers up to 17 kg mol^−1^ did not translate into a subsequent increase in performance, which could be due to limitations in the solubility and decreasing molecular mobility of longer polymer chains (this hypothesis was validated by experimenting with polymer batches of notably large molecular weight, the results of which are described in the Supporting Information). Besides, for the three polymers with the highest molecular weights, which were examined by CPE, we noted an undesirable increasing content of (7,5) chirality (Figure 3e–g). This was reflected in the calculated EFs, which were ≈1.71–1.98 regardless of the molecular weight of PFO‐BPy6,6′ for (6,5) chirality, while they increased for (7,5) SWCNTs from less than 0.20 to 0.49 when the polymers above 10 kg mol^−1^ were employed (Figure S6b, Supporting Information).

The CPE of SWCNTs relies on the reversible adsorption and desorption of polymer chains on SWCNTs during agitation (sonication or shear mixing). It is reasonable to assume that the high M_w_ batches may be challenging to desorb from unpreferred SWCNTs due to their low mobility and higher total energy of absorption. Consequently, they could not readily detach from the untargeted (7,5) SWCNTs to migrate to the desired (6,5) SWCNTs in the short time span of sonication, which was just 8 min. This problem can be counteracted by increasing the sonication time or temperature of the process mixture to enable polymer chains to reach their optimal arrangement on the SWCNTs surface. Please refer to Supplementary Information to find out more about this aspect.

**Figure 4:**
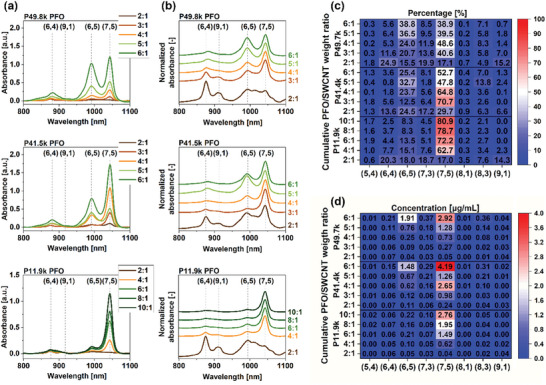
a) Raw and b) normalized optical absorbance spectra of SWCNTs suspended with P11.9, P41.5, and P49.8k PFO batches at various CP:SWCNT ratios. c) Percentage and d) concentrations of individual SWCNT species obtained by isolation with various PFO batches in a multistep procedure quantified by the deconvolution of corresponding optical absorbance spectra.

As the final component of this study, we investigated the combined effect of polymer molecular weight and conjugated polymer‐to‐SWCNT (CP:SWCNT) weight ratio on kinetics, yield, and the capacity for chiral discrimination in the context of selecting the optimal conditions for this process. The abovementioned conclusions were drawn from a single‐step CPE process using a predetermined CP:SWCNT ratio. To explore the highlighted aspects further, we developed a new procedure for repetitive CPE in which, after the single isolation sequence was finished, the supernatant (after its analysis using absorption spectroscopy) and the precipitate were recombined, a fresh portion of the polymer was added, and the entire mixture was again sonicated, ultracentrifuged, and reanalyzed. A detailed description of the devised methodology and a process diagram are available in the SI (Scheme S1, Supporting Information). For these experiments, we selected three batches of PFO (**Figure** **4**) and PFO‐BPy6,6′ (Figure S8, Supporting Information) of different molecular weights and evaluated their suitability for selective suspension of SWCNTs as a function of CP:SWCNT ratio. The CP:SWCNT ratio ranged from 2:1 to 10:1 for low‐molecular‐weight polymers (2:1 to 8:1 for PFO‐BPy6,6′) and from 2:1 to 6:1 (2:1 to 5:1 for PFO‐BPy6,6′) for moderate and high‐molecular‐weight batches. The scope of the CP:SWCNT ratios for PFO‐BPy6,6′ was reduced due to its lower solubility compared to PFO. This provided an opportunity to observe how the population of chiralities in the supernatant changed with a gradual increase in the concentration of polymer, as well as prolonged sonication time. The incremental addition of polymer followed by sonication each time also counteracted the undesirable aggregation processes that could occur when higher polymer loadings were used in a relatively low volume of solvent and the agitation time was short.

Analysis of the results for SWCNTs suspended with PFO displayed highly valuable trends. At the lowest CP:SWCNT ratio of 2:1, none of the examined PFOs showed appreciable SWCNT solubilization capability as the absorption spectra were of low intensity. However, starting from just 3:1 (or 4:1 for the shortest PFO), the dominant chirality in suspension was the expected (7,5). Moreover, for the polymer with the small MW, a consistent increase in selectivity toward wrapping (7,5) against (6,5) was evident with the increase in its content. On the other hand, PFOs of larger molecular weight did not provide such a level of (7,5) enrichment at increased CP:SWCNT ratios. Besides, even at low CP:SWCNT ratios, these long polymers produced more contaminated suspensions of (7,5) SWCNTs, with a notable presence of (6,5), (6,4), (9,1), and other species.

Therefore, in this multistep approach, PFOs with the highest MW and dispersity were unexpectedly of limited utility in the CPE sorting of SWCNTs since they produced suspensions of inferior quality. On the contrary, in the classical one‐step CPE process, long‐chain polymers with a CP:SWCNT ratio of 6:1 were preferred. This reveals that the homogenization and polymer addition schemes have a higher than anticipated importance in this process. What do these results tell us about the mechanism of the SWCNT selective isolation process?

It seems that in the case of PFOs, a minimum of four and preferably not less than six‐fold excess of polymer relative to SWCNTs is crucial to establish extraction selectivity. Interestingly, this excess needs to be provided from the beginning of the process to prevent the emergence of non‐selectively wrapped SWCNTs of other chiralities, which deteriorate the purity of the collected material (Figure 4c). Previous reports indicated that PFO likely forms a multilayer structure on the surface of SWCNTs.^[^ ^40^, ^41^ ^]^ Hence, it is reasonable to assume that a high initial CP:SWCNT ratio is needed to promote the deposition of multiple PFO macromolecules on the preferred (7,5) SWCNTs. Such conditions ensure that the abovementioned multilayer structure around the (7,5) SWCNTs is thick enough, so that the PFO/(7,5) SWCNTs hybrids can resist the ultracentrifugation step, stripping away the polymer molecules from the SWCNT surface. The necessity to establish a relatively high amount of PFO in the system from the beginning suggests that the adsorption of PFO on (7,5) SWCNTs is a self‐accelerating process. In this scenario, PFO macromolecules prefer to form another layer around this SWCNT type rather than deposit on different SWCNT species. The polymer macromolecules have a tendency to adsorb on various SWCNT species present in the raw material, but under vigorous sonication/shear mixing conditions, there is a constant adsorption/desorption exchange of polymer macromolecules on the SWCNT surface.^[^ ^42^ ^]^ Concomitantly, at high CP excess with respect to SWCNTs, the multilayer structures established around (7,5) SWCNTs are sufficiently extensive that it is energetically favorable for PFO to coordinate with other PFO species around this type of SWCNT, rather than coat the other non‐preferred SWCNT chiralities. The formation of multilayer polymer coating around SWCNTs in CPE is elaborated in the Supplementary Information file.

Short PFO chains, i.e., below 18 kg mol^−1^, have slightly different behavior, but they follow these principles (Figure 4c,d). In a single CPE process, short chains are unable to produce a layer with sufficient total interaction energy to survive the ultracentrifugation process. However, the successive addition of fresh portions of polymer gradually produces a multilayered structure on the fastest and most stably wrapped chirality, (7,5). In the case of polymer chains with short average lengths, the adsorption/desorption process is likely to be more rapid since they can establish fewer interactions with SWCNTs. Consequently, they are less stable on the SWCNT surface and can be more easily desorbed during centrifugation or storage. Therefore, even if they adsorb on non‐preferred species, due to their high mobility, they can easily end up on (7,5) SWCNTs upon a number of adsorption/desorption cycles caused by homogenization. Remarkably, such short polymers previously considered unsuitable for the classical CPE process provided similar purity levels and even higher yields in this novel multistep procedure with a gradual increase of PFO concentration, requiring only a slightly increased CP:SWCNT ratio of 10:1 (Figure 4c,d).

For the second polymer tested, i.e., PFO‐BPy6,6′, the effect of molecular weight on extraction selectivity was considerably weaker (Figure S8, Supporting Information). Only the polymer with the smallest weight, i.e., M_w_≈5.6 kg mol^−1^, underperformed at CP:SWCNT = 2:1. On the other hand, an increase in this ratio gave a gradual increase in the (6,5) concentration in the supernatant in all the examined polymers (Figure S8c, Supporting Information). The most capable polymer was P’9.9k with a moderate M_w_≈9.85 kg mol^−1^ and Đ≈1.61, as previously highlighted. Unexpectedly, employing multistage CPE with this polymer achieved an unprecedented near‐monochiral concentration of (6,5) with leading optical absorbance values of A(6,5)@S11 = 9.5, which was equivalent to ≈22 µg mL^−1^. The high concentration of the as‐generated suspension was well illustrated by a picture of this sample, wherein the suspension had a vivid and intense purple color (Figure S9, Supporting Information). This surpassed the concentration of the best sample obtained by means of classical single‐step CPE (Figure 3) by fourfold using a different polymer batch. Interestingly, it outperformed the same batch employed in the one‐step approach by more than a factor of six (Figure S8d, Supporting Information). These values stand out in comparison to existing literature data.^[^ ^20^, ^21^, ^43^ ^]^ The reported concentration range was two‐times higher than that of commercially available polychiral dispersions (with SWCNT concentration≈10 µg mL^−1^), the latter comprising up to 19 chiralities, rather than predominantly one type of SWCNT with highly defined characteristics.^[^ ^20^, ^43^, ^44^ ^]^

Besides reaching such a high performance, this result provided valuable insight into the mechanism of this process, confirming the observation already discussed in this paper that an upper limit of the optimal molecular weight range existed for PFO‐BPy6,6′. Therefore, at excessive CP:SWCNT ratios, the SWCNT solubilization yield decreased, which was most likely related to the intensification of the polymer self‐aggregation supported by the solubility limitations revealed by this study. This, in turn, promoted precipitation of the polymer and polymer‐SWCNT hybrids in the sediment after centrifugation. Interestingly, the lowest molecular weight polymer examined (P’5.6k) showed very similar activity (SWCNT solubilization efficiency) to the highest molecular weight polymer evaluated (P’17.4k) when CP:SWCNT compensation was carried out by increasing this ratio to 8:1. This is a crucial finding, as drastically different CP:SWCNT ratios are applied by researchers, even for the same polymer, which may limit or eliminate the suitability of this polymer for CPE of SWCNTs. Thus, optimization in this regard for a given polymer batch is highly advised.

As our research reveals, the implementation of multistage selective isolation methodology significantly enhances the sorting efficiency for all the tested PFO‐BPy6,6′. What is particularly important from the green chemistry perspective is that in such an approach, the atom efficiency of CP also increases at least several times. Notably, even for the smallest polymer (P’5.6k), doubling the total CP:SWCNTs ratio from 4:1 to 8:1 in the transition from a single to a multistep procedure resulted in a nearly 22‐fold increase in yield (from 0.51 to 11.23 µg mL^−1^). Similarly, for the highest molecular weight polymer evaluated by CPE (P’17.4k), we observed a three‐fold increase in yield with only a 25% increase in the amount of polymer. In the context of chirality‐oriented selectivity, the discrepancies are rather minor. PFO‐BPy6,6′ polymers with M_w_ < 10 kg mol^−1^ exhibited almost no difference in selectivity and isolated only residual amounts of chiralities other than (6,5) (Figure S8c, Supporting Information).

**Figure 5:**
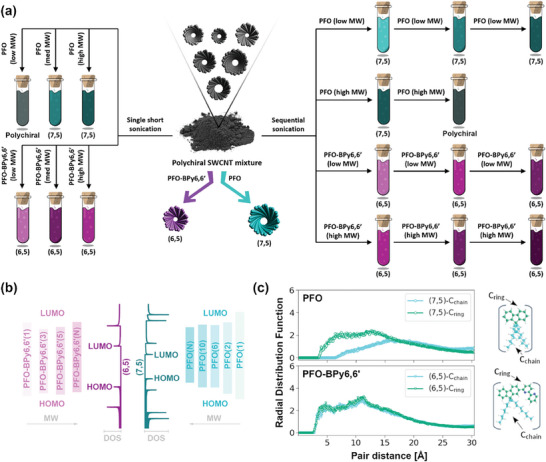
a) Visual overview of key findings regarding the isolation of (6,5) and (7,5) SWCNTs using PFO‐BPy6,6′ and PFO, respectively. b) Energy levels of (6,5) SWCNT, (7,5) SWCNT as well as indicated polymers used to suspend them. The number in brackets indicates the number of constituting units of an examined polymer. The density of states (DOS) for (6,5) and (7,5) SWCNT was taken from^[^ ^45^ ^]^ and aligned with the Fermi level calculated in this work. c) Radial Distribution Functions (RDFs) between SWCNT surface and polymer side chain carbon atoms (C_chain_) and conjugated polymer main chain carbon atoms (C_ring_) established for two units of (7,5) SWCNT interacting with ten units of PFO polymer and two units of (6,5) SWCNT interacting with five units of PFO‐BPy6,6′ polymer in toluene solution (1000 molecules). Schematic diagrams showing two distinct regions of both polymers: the main and the side chains are displayed next to the RDFs. All carbon atoms constituting polymer main chains are marked in teal, whereas carbon atoms forming side chains are cyan. Hydrogen and nitrogen atoms are marked in white and blue, respectively.

The visual representation of the main conclusions, which shed more light on the elusive mechanism of the CPE process, is provided below (**Figure** **5**). Overall, through tailoring of polymer characteristics and adjusting of the extraction conditions, one can obtain optically pure SWCNT suspensions of (6,5) and (7,5) SWCNTs, according to the acquired 2D photoluminescence excitation‐emission maps (Figure S10, Supporting Information). Finally, the remaining task was to justify the dissimilar behavior of PFO and PFO‐BPy6,6′, as shown above.

To accomplish this goal, we engaged spin‐polarized density functional theory (DFT) computations, molecular dynamics (MD) and time‐stamped force‐bias Monte Carlo (MC)‐simulations, which unraveled crucial differences between these two polymers (Figure 5b,c). First, based on the estimated HOMO and LUMO energy levels obtained at DFT/B3LYP/DZP theory level, it was clear that the bandgap of PFO was dependent on the molecular weight of the polymer, as it decreased considerably with the increase in the monomer unit (Figure 5b). On the other hand, the relationship between the polymer bandgap and its length was much less evident in the case of PFO‐BPy6,6′. This aspect may explain the exceptionally robust nature of PFO‐BPy6,6′, which was capable of extracting highly pure (6,5) SWCNT fractions regardless of the size of the used polymer. In contrast, extraction of monochiral (7,5) SWCNTs with PFO demanded polymers of high molecular weight. It should be stressed that establishing a proper alignment between the energy levels of the polymer and the SWCNTs is essential for the charge transfer to occur, which drives the CPE.^[^ ^46^ ^]^ Second, as MD/MC simulations employing full periodic table bonded valence forcefield showed (Figure S2, Supporting Information), these polymers tended to arrange differently with respect to SWCNTs (Figure 5c; Figure S11, Supporting Information). While PFO very much preferred to orient itself only with the main polymer chain facing the SWCNTs, PFO‐BPy6,6′ did not exhibit such an inclination, and it was equally probable to find the main‐ or side chain in the vicinity of SWCNTs. Moreover, note that both the main and side chains of PFO‐BPy6,6′ polymer were positioned considerably closer to the lateral surface of (6,5) SWCNT than the PFO main chain to the (7,5) SWCNT. The minimal distance between (6,5) SWCNT and PFO‐BPy6,6′ polymer main/side chain was smaller than 4 Å, while the minimal distance between (7,5) SWCNT and PFO main chain was larger than 6 Å. Consequently, the exceptionally high binding strength of PFO‐BPy6,6′ to SWCNTs could be justified by its capacity to wrap SWCNTs tightly. As the modeling results showed, it coats SWCNTs not only using the main polymer chain, but the alkyl side chains as well.

## 3 Conclusion

The present study fills a crucial gap in our understanding of the behavior of conjugated polymers in the chirality‐selective isolation of SWCNTs. Previous research relied on less common polymers, such as F8BT and PFDD, or non‐selective suspension using polythiophene.^[^ ^11^, ^12^ ^]^ The conducted research explains the substantial variations in results obtained from PFO‐based and PFO‐BPy6,6′‐based CPE reported by different research teams, a matter not yet addressed in the literature.

Our findings emphasize the challenges posed by PFO, requiring both a medium‐ or high‐molecular‐weight batch and a suitable methodology for effective use. Improper use may lead to misconceptions about its selectivity and efficiency, possibly contributing to its lower popularity compared to PFO‐BPy6,6′, which is commonly used to extract (6,5) SWCNTs. While optimizing the process methodology and selecting an adequate composition of the process mixture can effectively alleviate its drawbacks, this approach has been marginalized in the existing literature. With our revised methodology, isolation efficiencies of 3.0–4.0 µg mL^−1^ for (7,5) can be achieved, with an exceptional purity of ≈90%, for the first time reaching the performance of the most commonly used system, i.e., PFO‐BPy6,6′ in toluene, for the separation of (6,5) SWCNTs.^[^ ^20^, ^21^, ^43^ ^]^ In contrast, purification of SWCNTs using PFO‐BPy6,6′ shows a remarkable tolerance to macromolecular parameters, process mixture composition, and isolation methodology. Regardless of the extraction conditions employed, most of the generated fractions displayed appreciable (6,5) purity exceeding 90%. It should be emphasized, however, that its process performance is also subject to high variability in terms of efficiency. Skillful use of the advantages of this polymer yields dispersions with unprecedented concentrations of >20 µg mL^−1^. The modeling carried out highlighted the key differences between PFO and PFO‐BPy6,6′, which may justify their dissimilar behavior.

Summarizing the investigations performed and the conclusions drawn, we can establish general rules for sorting SWCNTs with conjugated polymers. First, a CP:SWCNT ratio of ≈4:1–6:1 usually allows the purest and most concentrated SWCNT dispersions to be obtained. Second, in the case of PFO, this excess can be increased to favor the formation of multilayers, increasing selectivity and sorting efficiency. Third, the utility of polymers with low molecular weight can be considerably increased by using them in the multistage isolation of SWCNTs. Fourth, in the case of polymers with high molecular weight, CP:SWCNT ratios can be lowered, but the sonication time and/or sonication temperature should be extended and/or increased to improve the molecular dynamics limited by the chains’ length. Moreover, it is noteworthy that the correlation between molecular weight and the physicochemical parameters, properties, and behavior of polymers in the liquid medium holds universal significance. This insight extends beyond our specific research and can be applied to other processes involving CP‐wrapped SWCNTs, such as excess polymer removal, film formation, or circuit printing using SWCNTs. Particularly in these applications, SWCNTs wrapped with low molecular weight polymers exhibit greater ease of use, facilitating straightforward removal through washing. Besides, due to the large number of polymer batches produced in this study, which was necessary to examine SWCNT sorting by CPE thoroughly, new knowledge was generated about the impact of synthesis parameters on the characteristics of the obtained materials.

Finally, our study holds crucial implications for the broad‐scale implementation of SWCNTs. With our methodology, dispersions of nearly 22 µg mL^−1^ for (6,5) SWCNT/PFO‐BPy6,6′ and up to 4.2 µg mL^−1^ for (7,5) SWCNT/PFO become easily achievable. This considerable increase in the concentration of the monochiral species translates into a reduction in the cost of generation of these highly valuable materials. Consequently, the results of this study allow them to be harnessed in R and D areas where costs were previously a significant barrier.

Further progress in this field hinges upon the elucidation of the mechanism of the CPE. MD modeling, especially in combination with time‐stamped force‐bias Monte Carlo (MC)‐simulations, is very useful for interpreting this process, facilitating the rational design of improved SWCNT sorting strategies using the CPE approach. However, reaching a full understanding of the interactions between all components of the system may necessitate additional first and/or second‐principle calculations of electronic properties, which are, unfortunately, relatively time‐ and resource‐intensive. Nonetheless, at the moment, it seems that only through multiscale modeling it may eventually become possible to predict how a polymer of a certain structure could extract a particular SWCNT type, which will unleash the full potential of CPE.

## 4 Experimental Section

All the information about the Experimental Section is given in Supporting Information.

## Conflict of Interest

The authors declare no conflict of interest.

## Supporting information

Supporting Information

## Data Availability

The data that support the findings of this study are available from the corresponding author upon reasonable request.
