# Supplementary material for: Size Matters in Conjugated Polymer Chirality‐Selective SWCNT Extraction
Source: Adv Sci (Weinh). 2024 May 24;11(29):2402176. doi: 10.1002/advs.202402176 (PMC11304282; doi:10.1002/advs.202402176)
Supplement: Supplementary file 1 — Supporting Information [file ADVS-11-2402176-s001.pdf]

ADVANCED SCIENCE
Open Access

Supporting Information

for *Adv. Sci.*, DOI 10.1002/advs.202402176

Size Matters in Conjugated Polymer Chirality-Selective SWCNT Extraction

Andrzej Dzienia, Dominik Just, Tomasz Wasiak, Karolina Z. Milowska, Anna Mielańczyk, Norman Labedzki, Sebastian Kruss and Dawid Janas**

Supporting information

Size Matters in Conjugated Polymer Chirality-Selective SWCNT Extraction

Andrzej Dzienia [a,*], Dominik Just [a], Tomasz Wasiak [a], Karolina Z. Milowska [b,c], Anna Mielańczyk [a], Norman Labedzki [d,e], Sebastian Kruss [d,e], Dawid Janas [a,*]

[a] Department of Chemistry, Silesian University of Technology, B. Krzywoustego 4, 44-100, Gliwice, Poland

[b] CIC nanoGUNE, Donostia-San Sebastián 20018, Spain

[c] Ikerbasque, Basque Foundation for Science, Bilbao 48013, Spain

[d] Department of Chemistry, Ruhr-University Bochum, 44801 Bochum, Germany

[e] Biomedical Nanosensors, Fraunhofer Institute for Microelectronic Circuits and Systems, 47057 Duisburg, Germany

* Corresponding authors: Andrzej.Dzienia@polsl.pl, Dawid.Janas@polsl.pl

Table of Contents

1. Materials 3
   1.1. Reagents for polymer synthesis 3
   1.2. Carbon nanotubes 3
2. Methods 3
   2.1. Polymer synthesis 4
   2.2. Preparation of SWCNT dispersions in a single-step CPE process 9
   2.3. Preparation of SWCNT dispersions in a multi-step CPE process 9
   2.4. Modeling details 11
      2.4.1. DFT calculations 11
      2.4.2. MD/MC calculations 13
3. Characterization 15
   3.1. Nuclear Magnetic Resonance (^{1}H NMR) 15
   3.2. Size Exclusion Chromatography (SEC) 15
   3.3. UV-VIS Spectroscopy 15
   3.4. Photoluminescence excitation-emission mapping 16
   3.5. Deconvolution of optical absorbance spectra 16
   3.6. Enrichment factor 16
   3.7. Raman Spectroscopy 17
   3.8. Atomic Force Microscopy 17
4. Results 18
   4.1. Confirmation of polymer structure by ^{1}H NMR 18
   4.2. Deconvolution of optical absorbance spectra 19
   4.3. Enrichment factors for single-step CPE process 20
   4.4. Determination of the structure of polymer coating around the isolated SWCNTs 21
   4.5. Composition and concentration of SWCNT suspensions generated using multi-step CPE process 23
   4.6. Photoluminescence excitation-emission mapping of SWCNTs solubilized using polymers synthesized in this study 24
   4.7. Modeling of SWCNT-polymer interactions 25
   4.8. The impact of the polymer composition on the capacity for sorting SWCNTs 27
   4.9. The impact of the processing conditions on the SWCNT sorting performance 29
   4.10. Comparison of single- and multi-step CPE 31
5. Literature 33

1. Materials

All chemical reagents and solvents were used as supplied, without additional purification or drying (except when stated otherwise). The purity of the reagents, along with the manufacturer and data allowing identification, can be found below.

1.1. Reagents for polymer synthesis

9,9-dioctyl-2,7-dibromofluorene (AmBeed, cat. number: A307700, CAS: 198964-46-4, purity: 97%), 6,6′-dibromo-2,2′-dipyridyl (Angene, cat. number: AG0034BD, CAS: 49669-22-9, purity: 97%), 9,9-dioctylfluorene-2,7-bis(boronic acid pinacol ester) (Angene, cat. number: AG0034EZ, CAS: 196207-58-6, purity: 98%), Aliquat 336 TG (Alfa Aesar, cat. number: A17247, CAS: 63393-96-4, purity N/A), tetrakis(triphenylphosphine)palladium – $Pd(PPh_3)_4$ (Apollo Scientific, cat. number: OR4225, CAS: 14221-01-3, purity: >99%).

1.2. Carbon nanotubes

The study was carried out using (6,5)-enriched CoMoCAT SWCNTs (Sigma Aldrich, product number: 773735, lot: MKCR4865, purity: 95%-carbon basis, mean length of 1.09 µm determined by AFM).

2. Methods

One of the reasons why the influence of the macromolecular parameters of conjugated polymers on the selective isolation of CPE has not been understood so far is the considerable difficulty in obtaining materials with the desired properties. Until recently, known methods for the polycondensation of AB and AA/BB monomers have been highly uncontrolled [1] and thus have not allowed for the direct correlation of the composition of the reaction mixture and/or reaction time with the average molecular weight obtained. The development of controlled polymerization methods (so-called living polymerization) is one of the major points of interest in modern polymer chemistry [2]. It mainly refers to chain-growth polymerization, where the propagation of the polymer chain occurs via the active center [3,4]. Living character is achieved by eliminating or suppressing the termination reaction and controlling the number and reactivity of the active centers. The highly unified growth rate of the propagating chains, as well as the minimization of the permanent deactivation

phenomenon, translate into a reduction in dispersity and the establishment of a correlation between conversion and average molecular weight. However, polycondensation proceeds via a mechanism of step-growth, providing no control over the number of active centers [5]. Despite this limitation, the first pioneering papers have recently appeared that reveal the possibility of achieving a high level of control over the Suzuki polycondensation process [3,4]. Unfortunately, this comes at the cost of high synthetic requirements, typically of a multistep character, which eliminates the prospective scalability of such production. To avoid these issues, we used a classical Suzuki coupling, synthesized multiple batches of polymers of diverse molecular characteristics, and then examined their application to sorting SWCNTs using CPE.

2.1. Polymer synthesis

The process of PFO and PFO-BPy6,6' synthesis was carried out according to the general Suzuki coupling procedure (Figure S1).

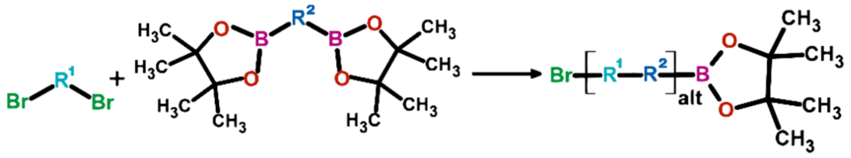

Figure S1 Schematic diagram of Suzuki Polycondensation (SPC).

In general, the boronic ester, i.e., 9,9-dioctylfluorene-2,7-diboronic acid bis(pinacol)ester (0.5 g, 0.763 mmol, 1 eq.), and the appropriate amount of dibromo derivative were added to a high-pressure glass reactor vessel. For the synthesis of polydioctylfluorene (PFO), a homopolymer, the second monomer was 9,9-dioctyl-2,7-dibromofluorene (0.418 g, 0.763 mmol, 1 eq.) and for poly[(9,9-dioctylfluorene-2,7-diyl)-*alt*-(6,6'-{2,2'-bipyridine})] (PFO-BPy6,6'), a co-polymer, it was 6,6'-dibromo-2,2'-bipyridine (0.240 g, 0.763 mmol, 1 eq).

The reactor was then filled with 1M Na_2CO_3 solution (14 mL) and toluene (14 mL). Finally, three drops of Aliquat 336 phase transfer catalyst (PTC) were added. The mixture was flushed with argon for 30 min, and $Pd(PPh_3)_4$ (0.20 g, 0.17 mmol, 0.23 eq.) was added. Subsequently, the reaction mixture was vigorously stirred at 85°C for 3 days for PFO or 5 days for PFO-BPy6,6'. After cooling down, the reaction mixture was diluted with 500 mL of chloroform and washed three times with 150 mL of water. The resulting organic layer was

evaporated to dryness and then dissolved in 50–100 mL of chloroform, in order to obtain a low-viscosity liquid that would produce small particles or thin fibers when precipitated. The final product was precipitated from a combination of methanol and water (in a ratio of 9:1), filtered, and subjected to two washes with 100 mL of cold methanol, followed by two additional washes with 100 mL of cold acetone. Typically, after one day of drying, which was necessary to reach a constant weight, yellow to green-gray powder, flakes, or fibers were obtained with a yield of 60–90% for PFO or white or beige to gray powder, flakes, or fibers with a yield of 40–80% for PFO-BPy6,6’. These materials were collected using a cellulose filter.

The purification process of PFO-BPy6,6' with moderate to high molecular weights should be modified due to its limited solubility in chloroform and toluene. The preferred solvent in terms of solubility is chlorobenzene. However, it has a high boiling point, which makes the concentration process difficult. The solution may be to use hot chloroform or toluene. In the case of PFO-BPy6,6' with M_w = 17.4 kg/mol, the limiting achievable concentration in toluene is 1.3 - 2 mg/mL. While the batch with M_w = 98.5 kg/mol reached a solubility of less than 0.25 mg/mL and required heating to 40-50°C with the help of sonication. The same polymer achieved solubility in the range of 1.3 - 2 mg/mL in chlorobenzene. On the other hand, PFO with high molecular weights, i.e., M_w = 81.7 kg/mol, reached concentrations of more than 5 mg/mL in toluene without the need for intense heating or extended sonication.

In order to adjust the purification process of the reaction mixture of high molecular weight PFO-BPy6,6', it is recommended to remove the aqueous phase of the reaction mixture with a separatory funnel after the reaction and pour a portion of pure water over the organic phase. Then, the biphasic mixture should be stirred vigorously on a magnetic stirrer with the heating temperature adjusted to the organic solvent used. After several hours of stirring, the aqueous phase is again removed, and the organic phase is concentrated. In the case of PFO-BPy6,6' with high molecular weights, precipitation can be carried out without considerable loss from fairly dilute hot chloroform. The volume of the methanol/water mixture should be adjusted to the volume of chloroform so that a layer of undissolved chloroform does not form at the bottom of the vessel.

To prepare a cross section of conjugated polymers with a wide range of molecular weights, the standard procedure was modified in a few particular aspects: the catalytic system

($Pd(PPh_3)_4$ or PdNPs/NiNWs), the reaction system activation method (thermal or microwave), the reaction temperature, the polymerization scale, the molar ratio of A:B monomers, the polymerization time, and type of base used. The influence of most of the above factors on the macromolecular parameters of the synthesized polymers has already been widely studied in the literature [6]. However, the application of the advantages of microwave radiation and the developed novel catalytic systems was immensely helpful as it expanded the range of achievable molecular weights, while simultaneously reducing the duration of a single synthesis substantially. The description of the methodology for carrying out SPC reactions using microwave radiation and the fabrication of catalysts based on nickel nanowires decorated with palladium nanoparticles was described in our previous publication [7]. A detailed list of the conditions used to obtain the various batches, along with their macromolecular parameters, can be found in Table S1.

Table S1 A list of the obtained polymers with their nomenclature and indication of the method of synthesis employed, as well as their macromolecular parameters determined by GPC.

Polymer	Batch name	M_w [kg/mol]	M_n [kg/mol]	Đ	Synthethic methods	Heating protocol	A:B ratio	Reaction scale (A monomer)	Temperature [°C]	Time [h]	Catalyst	Base
PFO	P3.1k	3.05	2.2	1.41	Microwave	Standard	1:1	83 mg	110	1	Pd/NiNWs	1M Na_2CO_3
	P4.0k	4.00	3.1	1.27	Microwave	Standard	1:1	83 mg	80	1	Pd/NiNWs	1M K_2CO_3
	P4.6k	4.60	3.5	1.33	Microwave	SPS	1:1	83 mg	80	1	Pd/NiNWs	1M K_2CO_3
	P6.6k	6.60	4.6	1.45	Microwave	Standard	1:1	83 mg	130	1	Pd/NiNWs	1M K_2CO_3
	P6.7k	6.70	5.0	1.35	Microwave	SPS	1:1	83 mg	130	1	Pd/NiNWs	1M Na_2CO_3
	P7.4k	7.40	5.4	1.36	Microwave	SPS	1:1	249 mg	110	1	Pd/NiNWs	1M Na_2CO_3
	P8.5k	8.45	5.2	1.61	Microwave	Standard	1:1	83 mg	110	1	Pd/NiNWs	1M K_2CO_3
	P8.7k	8.65	6.2	1.40	Microwave	SPS	1:1	83 mg	125	1	Pd/NiNWs	1M K_2CO_3
	P9.5k	9.50	6.2	1.53	Microwave	SPS	1:1	83 mg	110	1	Pd/NiNWs	1M K_2CO_3
	P11.3k	11.30	8.1	1.40	Microwave	Standard	1:1	83 mg	130	1	Pd/NiNWs	1M Na_2CO_3
	P11.9k	11.90	6.6	1.80	Microwave	SPS	1:1	83 mg	110	1	Pd/NiNWs	1M K_2CO_3
	P12.7k	12.65	8.4	1.51	Microwave	SPS	1:1	83 mg	110	1	Pd/NiNWs	1M Na_2CO_3
	P16.3k	16.25	8.3	1.96	Microwave	SPS	1:1	83 mg	110	1	Pd/NiNWs	1M Na_2CO_3
	P16.5k	16.45	10.5	1.57	Microwave	SPS	1:1	83 mg	110	1	Pd/NiNWs	1M Na_2CO_3
	P18.0k	18.00	7.7	2.35	Microwave	Power Cycling	1:1	83 mg	110	1	Pd/NiNWs	1M Na_2CO_3
	P25.7k	25.65	11.6	2.22	Thermal	Heating Bath	1:1	450 mg	85	72	$Pd(PPh_3)_4$	1M Na_2CO_3
	P26.0k	26.00	14.3	1.82	Microwave	SPS	1:1	83 mg	110	1	Pd/NiNWs	1M Na_2CO_3
	P28.0k	28.00	7.8	3.59	Microwave	Power Cycling	1:1	83 mg	110	1	$Pd(PPh_3)_4$	1M Na_2CO_3
	P29.2k	29.15	11.0	2.65	Thermal	Heating Bath	1:1	400 mg	85	72	$Pd(PPh_3)_4$	1M Na_2CO_3
	P41.2k	41.20	13.0	3.18	Thermal	Heating Bath	1:1	200 mg	85	72	$Pd(PPh_3)_4$	1M Na_2CO_3
	P41.5k	41.45	15.7	2.64	Thermal	Heating Bath	1:1	1000 mg	85	72	$Pd(PPh_3)_4$	1M Na_2CO_3
	P49.8k	49.75	15.6	3.19	Thermal	Heating Bath	1:1	250 mg	85	72	$Pd(PPh_3)_4$	1M Na_2CO_3
	P53.4k	53.35	23.5	2.27	Microwave	SPS	1:1	400 mg	110	1	$Pd(PPh_3)_4$	1M Na_2CO_3

Polymer	Batch name	M_w [kg/mol]	M_n [kg/mol]	Đ	Synthethic methods	Heating protocol	A:B ratio	Reaction scale (A monomer)	Temperature [°C]	Time [h]	Catalyst	Base
PFO-BPy6,6’	P’5.6k	5.55	3.5	1.59	Thermal	Heating Bath	1:1	500 mg	85	120	$Pd(PPh_3)_4$	1M Na_2CO_3
	P’6.0k	6.00	3.8	1.60	Thermal	Heating Bath	1:0.9	250 mg	85	96	$Pd(PPh_3)_4$	1M Na_2CO_3
	P’6.5k	6.55	4.2	1.56	Thermal	Heating Bath	1:1.1	250 mg	85	96	$Pd(PPh_3)_4$	1M Na_2CO_3
	P’6.6k	6.60	3.9	1.69	Thermal	Heating Bath	1:0.9	250 mg	85	120	$Pd(PPh_3)_4$	1M Na_2CO_3
	P’6.7k	6.65	4.5	1.47	Thermal	Heating Bath	1:0.9	250 mg	85	96	$Pd(PPh_3)_4$	1M Na_2CO_3
	P’7.0k	7.00	4.3	1.63	Thermal	Heating Bath	1:0.99	250 mg	85	120	$Pd(PPh_3)_4$	1M Na_2CO_3
	P’7.9k	7.85	5.5	1.43	Thermal	Heating Bath	1:1.1	250 mg	85	96	$Pd(PPh_3)_4$	1M Na_2CO_3
	P’8.7k	8.70	6.2	1.41	Thermal	Heating Bath	1:1.1	250 mg	85	120	$Pd(PPh_3)_4$	1M Na_2CO_3
	P’9.5k	9.45	7.2	1.31	Thermal	Heating Bath	1:0.9	250 mg	85	72	$Pd(PPh_3)_4$	1M Na_2CO_3
	P’9.9k	9.85	6.1	1.61	Thermal	Heating Bath	1:1.1	250 mg	85	144	$Pd(PPh_3)_4$	1M Na_2CO_3
	P’10.3k	10.25	6.3	1.64	Thermal	Heating Bath	1:0.9	250 mg	85	144	$Pd(PPh_3)_4$	1M Na_2CO_3
	P’10.6k	10.55	5.8	1.83	Microwave	Power Cycling	1:1	83 mg	110	1	Pd/NiNWs	1M Na_2CO_3
	P’11.7k	11.65	8.3	1.40	Thermal	Heating Bath	1:1.1	250 mg	85	72	$Pd(PPh_3)_4$	1M Na_2CO_3
	P’13.0k	12.95	8.6	1.51	Microvawe	SPS	1:1	83 mg	100	1	$Pd(PPh_3)_4$	1M Na_2CO_3
	P’13.7k	13.70	8.7	1.57	Thermal	Heating Bath	1:1.1	250 mg	95	72	$Pd(PPh_3)_4$	1M Na_2CO_3
	P’17.4k	17.35	11.7	1.48	Microwave	SPS	1:1	83 mg	100	1	$Pd(PPh_3)_4$	1M Na_2CO_3
	P’41.2k	41.10	13.4	3.07	Microwave	SPS	1:1	500 mg	100	0.75	$Pd(PPh_3)_4$	1M Na_2CO_3
	P’98.5k	98.50	35.0	2.82	Microwave	SPS	1:1	1000 mg	100	0.75	$Pd(PPh_3)_4$	1M Na_2CO_3
	P’118.1k	118.10	50.4	2.34	Microwave	SPS	1:1	1500 mg	100	0.75	$Pd(PPh_3)_4$	1M Na_2CO_3

Heating protocol:

- Standard – this protocol used variable power to obtain the target temperature and maintained it at approx. ± 1°C. The standard protocol provided a comparable intensity of microwave radiation.
- SPS – this mode kept the power constant, which led to a higher temperature hysteresis ($\Delta T \approx 5°C$). During the process, SPS mode power was delivered in pulses.
- Power Cycling – this mode was similar to SPS, but instead of a heating time, the number of heating cycles was set. Each cycle was characterized by a higher temperature hysteresis, $\Delta T \approx 30°C$. The number of cycles was adjusted to maintain a time of 1h.

2.2. Preparation of SWCNT dispersions in a single-step CPE process

To screen the suitability of various synthesized polymer batches for the sorting of SWCNTs, a standard suspension process was used. In this approach, 1.5 mg of the selected SWCNTs and 9 mg of PFO or 6 mg of PFO-BPy6,6' were weighed into separate 15 mL glass vials. Subsequently, the polymer was dissolved in 4.5 mL of toluene, and the resulting polymer solution was then transferred to a vial containing the pre-weighed SWCNTs. The mixture underwent a preliminary sonication step in an ice-cooled sonicator bath for 30 minutes (Polsonic, Sonic-2, 250 W). The subsequent crucial step involved the main sonication, utilizing a tip sonotrode (Hielscher UP200St ultrasonic generator), which operated at a power of 30 W for 8 minutes, maintaining the temperature of the processed mixture close to 5°C. Upon completion of sonication, the thick suspension was carefully transferred to 10 mL conical tubes and underwent centrifugation at 10,000 rpm (15,314 × g) for 5 minutes to eliminate bundled SWCNTs and polymer aggregates. Finally, 90% of the resulting supernatant was meticulously transferred to a fresh vial for subsequent spectroscopic analysis.

2.3. Preparation of SWCNT dispersions in a multi-step CPE process

To investigate how the change in CP:SWCNT ratio affects the composition of the extracted SWCNTs, a multistep CPE approach was devised, which enabled the activity of a new polymer to be investigated more quickly and cost-effectively. As we demonstrated in this manuscript, this approach may also be used to manipulate the performance and selectivity of specific polymer batches, which, under classical conditions, do not deliver the desired results. In the case of a relatively high CP:SWCNT ratio, only a fraction of the supplied polymer participates in the wrapping of individual SWCNTs. The rest exists in an unbound form in the dispersion or post-process sediment [8]. Even though only a part of the polymer participates in effective wrapping, reducing the polymer excess often results in a deterioration in process efficiency and/or selectivity. This is particularly evident in the case of PFO, which does not bind as strongly to SWCNTs as PFO-BPy, therefore, optimization of its content is even more essential.

For the multistep process, the precipitate and supernatant, produced after sonication and centrifugation, were recombined after analysis by spectroscopic methods. To ensure a

complete transfer of the precipitate from the centrifuge flask to the glass vial used for sonication, a part of the supernatant was initially used to suspend the precipitate. Then, after shaking, the diluted precipitate was transferred to the previously used glass vial. The rest of the supernatant was used to quantitatively transfer the remaining amount of non-suspended precipitate. Once both the precipitate and supernatant were reunited, a pre-weighed fresh portion of the polymer was introduced. The process mixture thus obtained was sonicated again using a sonotrode, followed by centrifugation and another separation into precipitate and supernatant probed by spectroscopy. Every two purification sequences, about 10% of the initial volume of fresh solvent was added to the process solution to account for solvent loss during sonication.

Both methodologies are visualized in Scheme S1ab. The multi-stage CPE approach was redesigned (Scheme S1b) compared with the state-of-the-art [9] (Scheme S1c). After each sonication stage, the supernatant was taken away only for spectroscopic analysis, but then it was recombined with the sediment for the subsequent round of sonication/centrifugation. Secondly, the concentration of polymer was gradually increased in the system. The goal was to observe how a successive increase in the polymer concentration on the surface of a known amount of SWCNTs affected the capacity of the system for sorting polychiral SWCNT mixtures.

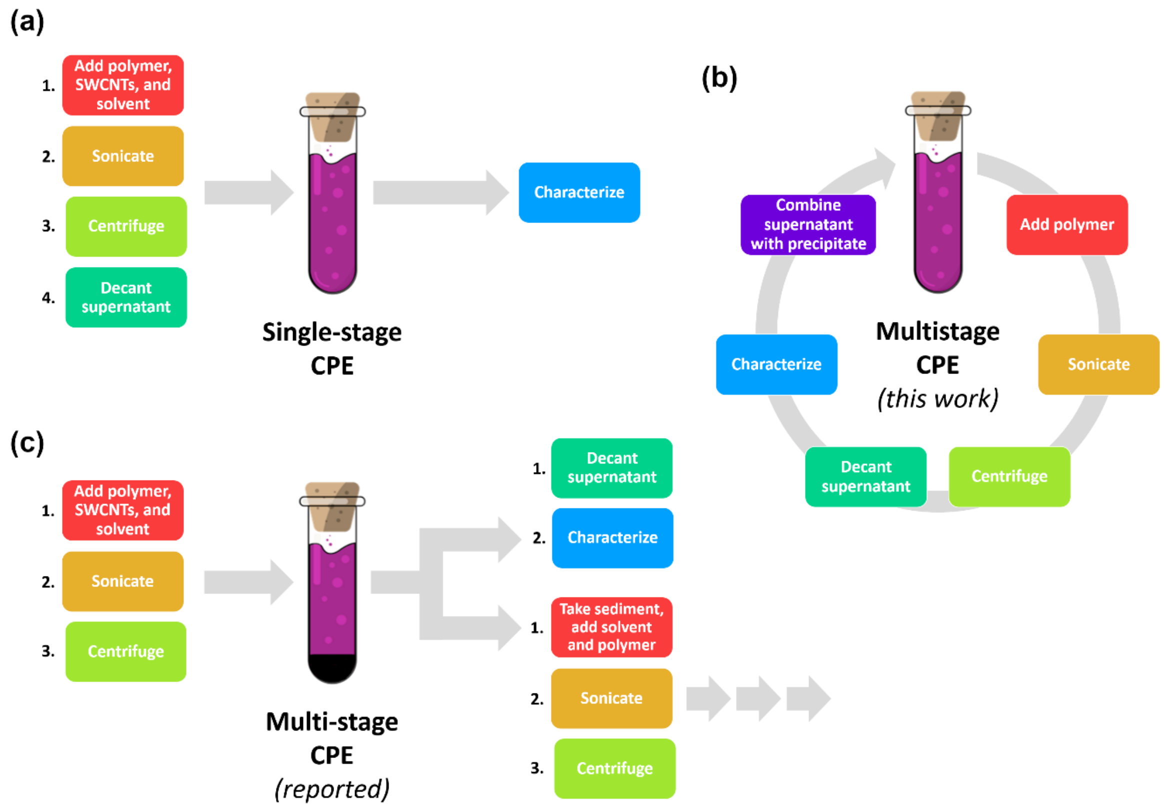

Scheme S1 (a) Single- and (b) Multi-stage CPE approaches used in this work. (c) Previously reported multi-stage CPE procedure.

For instance, the results visualized in Figure 4 should be understood as follows. The polymer (P49.8k PFO) was combined with SWCNTs in toluene in a 2:1 ratio, the mixture was sonicated for 8 minutes, the supernatant was collected, analyzed, and recombined with the precipitate. Subsequently, the ratio of PFO to SWCNTs was increased to 3:1, and the process was repeated until reaching the polymer to SWCNT ratio of 6:1. Characterization of the obtained material by AFM showed no statistically significant change in SWCNT length after the multi-step processing.

2.4. Modeling details

2.4.1. DFT calculations

The spin-polarized Density Functional Theory (DFT) [10,11] calculations of (6,5) and (7,5) SWCNTs, PFO and PFO-BPy6,6’ polymers of different length (PFO: 1, 2, 6, 10 or infinite number of monomers; PFO-BPy6,6’: 1, 3, 5 or infinite number of monomers) were carried out in generalized gradient approximation (GGA) employing hybrid non-local exchange and correlation functional – B3LYP [12–15] and double-ζ plus polarization numerical basis (DZP)

sets, as implemented in QuantumATK [16,17]. Previous studies have shown that B3LYP functional performs reasonably well for predicting the electronic structure of different nanostructures [18–21]. The DFT calculations of both finite polymers, were performed in 'molecule configuration' mode without periodic boundary conditions applied. The Brillouin zone was sampled only in the Γ point, while the density mesh cut-off for real-space integrals was set at 300 Ry. In case of infinite polymers and SWCNTs, calculations were carried out in 'bulk configuration' mode with 1D periodic boundary conditions applied. Consequently, the sampling of the Brillouin zone was increased to (1 × 1 × 3) and (1 × 1 × 7) k-points in Monkhorst and Pack scheme [22], respectively. All structures were relaxed until the maximum force acting on any atom was lower than 0.004 eV/Å and the maximum stress changed by less than 0.1 GPa. The self-consistent field (SCF) cycle was iterated until the total energy changed by less than 10^{-6} Ha, and the density matrix elements by less than 10^{-6} per iteration. The solvent was not included in those calculations. It is not common practice to consider solvent in spin-polarized DFT calculations employing hybrid exchange-correlation functional of neutral molecules as such calculations are very computationally demanding. Preliminary results showed no significant changes in the polymer structures in the presence of the solvent after geometry optimization.

2.4.2. MD/MC calculations

To investigate the interactions between PFO-BPy6,6' and (6,5) SWCNT or between PFO and (7,5) SWCNT, we performed series of molecular dynamics (MD) and time-stamped force-bias Monte Carlo (MC) [23,24] simulations of infinite SWCNTs interacting with polymers in toluene (Figure ST1). Due to applied 3D periodic boundary conditions, simulation boxes contained only 2 units of (6,5) or (7,5) SWCNT. Simulations boxes with dimensions sufficient to avoid direct interactions between SWCNT-polymer complex images (4.6 nm, 4.6 nm and 8.2 nm for (6,5) SWCNT or 8.9 nm for (7,5) SWCNT along X, Y, and Z directions) were filled with toluene (1000 molecules) using Packmol [25].

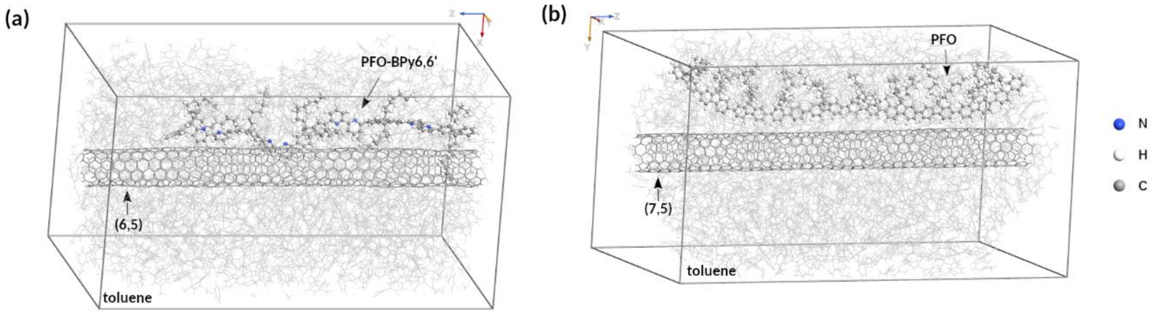

Figure S2 Snapshots of the final configurations for the simulation boxes containing (a) two units of (6,5) SWCNT interacting with PFO-BPy6,6' (five repeatable units) or (b) two units of (7,5) SWCNT interacting with PFO polymer (ten repeatable units) suspended in toluene (1000 molecules). 3D periodic boundary conditions were applied, making SWCNTs effectively infinite. SWCNTs, solvent, and polymer molecules are represented using stick, line, and ball-and-stick models, respectively. For clarity, solvent molecules are drawn with a higher degree of transparency than other system components.

The MD/MC simulations were performed as follows:

- Both systems were pre-optimized (500 000 steps) using FIRE algorithm [26].
- MD simulations were first carried out in NVT ensemble employing a Berendsen thermostat [27] at 300 K. Random initial velocities of all atoms were assigned according to the Maxwell-Boltzmann distribution. The relaxation time of the thermostat was set to 100 fs, and the simulations were carried out with a time-step of 0.01 fs over 0.5 ps (5,000 steps). These simulations were followed by 0.5 ps (50,000 steps) NPT simulations at 300 K and 1 bar employing Berendsen thermostat and barostat. The thermostat and barostat relaxation times were set to 100 fs and 500 fs, respectively. The time step was kept the same as for previous simulations. The estimated compressibility of the system relating volume changes to pressures

changes was set to 0.0001 bar^{-1}. Then, the Martyna-Tobias-Klein [28] barostat and thermostat were used for 20 ps (2 000 000 steps) NPT simulations, employing the same parameters.

- Next, models were optimized (20 000 steps) using LBFGS algorithm [29] and further equilibrated in microcanonical (NVE) ensemble with a time-step of 0.1 fs over 5 ps (50 000 steps) at 300K and again optimized using FIRE (2 000 steps) and LBFGS (1500 steps) algorithms.
- NVE equilibration was repeated over 0.2 ns (500 000 steps), using time step of 0.2 fs, and followed by additional geometry optimization using FIRE (20 000 steps) and LBFGS (5000 steps) algorithms. To investigate the interactions between polymer and SWCNT on longer timescales, the MC simulations were performed subsequently. They were carried for 5.31139 ns (4 000 000 steps) at 300 K and 1 bar. During MC simulations, the maximum atom displacement was set to 0.05 Å, the estimated compressibility of the system related to volume changes to pressure changes was set to 0.0005 bar^{-1}, and the barostat factor was set to 1000. MC simulations were followed by additional 5 ns (10 000 000 steps) equilibration (NVE simulations) with time step of 0.5 fs at 300 K.

MD/MC calculations were performed using a full periodic table bonded valence forcefield - an Universal Force Field (UFF) potential [30], as implemented in QuantumATK [17,31]. Energy contributions to the UFF potential were represented by simple functions based on bond lengths, bond angles, torsion angles, inversion angles, and inter-atomic distances. The electrostatic interactions were calculated using smooth-particle-mesh-Ewald (SPME) solver [32]. The cut-off used for calculating the real-space interactions was set to 7.5 Å, while the relative accuracy of SPME summation to 0.0001. Atomic partial charges on each atom were assigned using QEq charge equilibration method [33]. Dispersive interactions were included in the form of Lennard-Jones potential [34–36] with 10 Å cut-off and 2 Å smoothing length. Radial distribution and angular distribution functions were calculated using the data obtained during last 1 ns of NVE simulations.

3. Characterization

Multiple batches of polymers were produced and characterized. The structures of the polymers were confirmed by ^1H NMR spectroscopy. Macromolecular parameters were assessed by GPC/SEC and are listed in Figure 2c and Figure 3c for PFO and PFO-BPy6,6', respectively. The obtained SWCNT dispersions using the synthesized polymers were analyzed using absorption and photoluminescence spectroscopy.

3.1. Nuclear Magnetic Resonance (^1H NMR)

The proton Nuclear Magnetic Resonance (^1H NMR) spectra of the obtained polymers were registered using a Varian Unity Inova spectrometer functioning at 400 MHz with $CDCl_3$ as the solvent. The ^1H chemical shifts were recorded in δ (ppm), utilizing the residual peak of chloroform-d at δ 7.26 as a reference. Standard experimental conditions were applied. The NMR spectra of examples of the synthesized polymers interpreted using the Bruker program are included below (Figure S3 and Figure S4).

3.2. Size Exclusion Chromatography (SEC)

Molecular weights and dispersity (Đ) indices were determined through Size Exclusion Chromatography (SEC) employing an Agilent 1260 Infinity system (Agilent Technologies), which was equipped with an isocratic pump, autosampler, degasser, thermostatic box for columns, and a differential refractometer, MDS RI Detector. Data collection and processing were performed using Agilent Technologies' Addon Rev. B.01.02 data analysis software. SEC-calculated molecular weights were derived from calibration using linear polystyrene standards ranging from 580 to 300,000 g/mol. The separation process involved a pre-column guard (5 µm, 50 × 7.5 mm) and two columns, namely PLGel 5 µm MIXED-C (300 × 7.5 mm) and PLGel 5 µm MIXED-D (300 × 7.5 mm). Measurements were conducted using chloroform (HPLC grade) as the solvent at a temperature of 30 °C and a flow rate of 0.8 mL/min.

3.3. UV-VIS Spectroscopy

Optical absorbance spectra were recorded (400 to 1100 nm) using a Hitachi U-2910 spectrophotometer. A double-beam mode was used with a pure solvent cuvette placed in the reference channel. Where indicated, spectra were normalized to the global minimum

between 600 and 900 nm to facilitate comparison between samples, which is a standard procedure [37].

3.4. Photoluminescence excitation-emission mapping

Excitation-emission photoluminescence maps (PL) were acquired using a ClaIR microplate reader (Photonetc, Canada). The data were registered in the ranges of 480–900 nm (excitation) and 900–1600 nm (emission). The results were then visualized using OriginPro 2022 software.

3.5. Deconvolution of optical absorbance spectra

Analysis of PL excitation-emission maps provides a convenient way to identify the presence of individual chiralities in the studied material. However, quantification of the obtained results is challenging due to issues such as fluorescence quenching induced by various factors or the dependence of photoluminescence quantum yield (PLQY) on chirality [38]. On the other hand, optical absorbance spectra are easily accessible, and less susceptible to such issues, but more demanding to interpret in order to estimate the composition of studied dispersions.

To this end, the obtained spectra were deconvoluted using the PTF Fit application [39]. Firstly, the baseline was subtracted using a function developed by Nair et al. [40] integrated into the program. Deconvolution was then performed using the Voigt function to reconstruct the spectra as a combination of individual SWCNT peaks. The obtained data enabled the estimation of the composition of the SWCNT suspensions and the concentrations of individual SWCNT species. Due to the inherent limitations of this quantification method related to the challenging nature of the deconvolution of complex spectra, the provided purity values are likely underestimated.

3.6. Enrichment factor

Typically, SWCNT sorting selectivity is determined as a measure of the share of a particular type of SWCNTs in the suspension with respect to other detected SWCNT types, as presented in Figures 2f and 3f. However, for a more comprehensive evaluation, considering the Enrichment Factor (EF) can offer greater insight. The EF takes into account (a) the abundance of a particular SWCNT type in the raw material, (b) the capabilities of a specific

polymer for the dispersion of SWCNTs, i.e., how concentrated an SWCNT suspension it produces. Hence, the EF can be computed as the ratio of the concentration of the selected chirality (C_i) to the total concentration of all SWCNTs in the dispersion (C_{all}). This ratio is then normalized against the corresponding ratio for a reference sample ($C_{i,REF}/C_{All,REF}$). This relation is summarized in the following equation:

$$EF = \frac{C_i/C_{All}}{C_{i,REF}/C_{All,REF}},$$

where C_i and $C_{i,REF}$ are the concentration of the selected i-chirality in the analyzed dispersion and reference sample, respectively, while C_{All} and $C_{All,REF}$ are the total concentrations of all registered SWCNT chiralities in the analyzed dispersion and reference materials, respectively.

Since the data for the reference are provided by a non-selective SWCNT dispersant, the calculated EF provides accurate information about the sorting selectivity. This is crucial because, in many instances, research is conducted on previously enriched material (e.g., (6,5)- or (7,6)-enriched CoMoCAT SWCNTs), which inherently contains a predominance of specific SWCNT types. In such cases, isolating more abundant SWCNTs is comparatively easier than those with significantly lower shares. Additionally, acknowledging the influence of the final concentration of sorted SWCNTs is imperative, as a higher concentration in the final material is desirable for better outcomes.

3.7. Raman Spectroscopy

Raman spectra were collected using an inVia Raman Ramascope with an excitation wavelength of 532 nm. Multiple spectra from different positions of the samples were recorded and averaged for each sample to ensure the statistical significance of the recorded data.

3.8. Atomic Force Microscopy

AFM images were registered using Bruker Nanowizard 5. For the imaging, the samples were immobilized on freshly cleaved mica. The determination of the SWCNT length and diameter of the polymer/SWCNT hybrids necessitated purification of the material to remove excess polymer. In this process, the suspensions created by CPE, i.e., PFO-BPy6,6'/SWCNTs (4:1) and PFO/SWCNTs (6:1), were centrifuged thrice, and, each time, after the generation of the precipitate, the supernatant containing polymer molecules was removed, and the fresh solvent was added.

4. Results

4.1. Confirmation of polymer structure by ^1H NMR

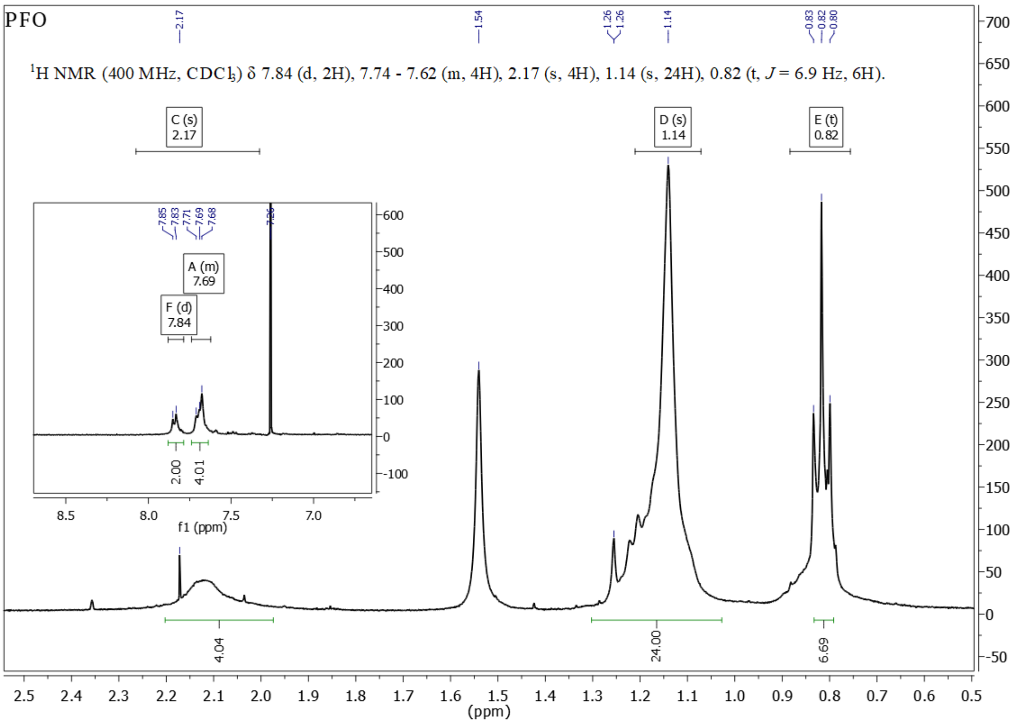

Figure S3 ^1H NMR spectrum of PFO synthesized in-house.

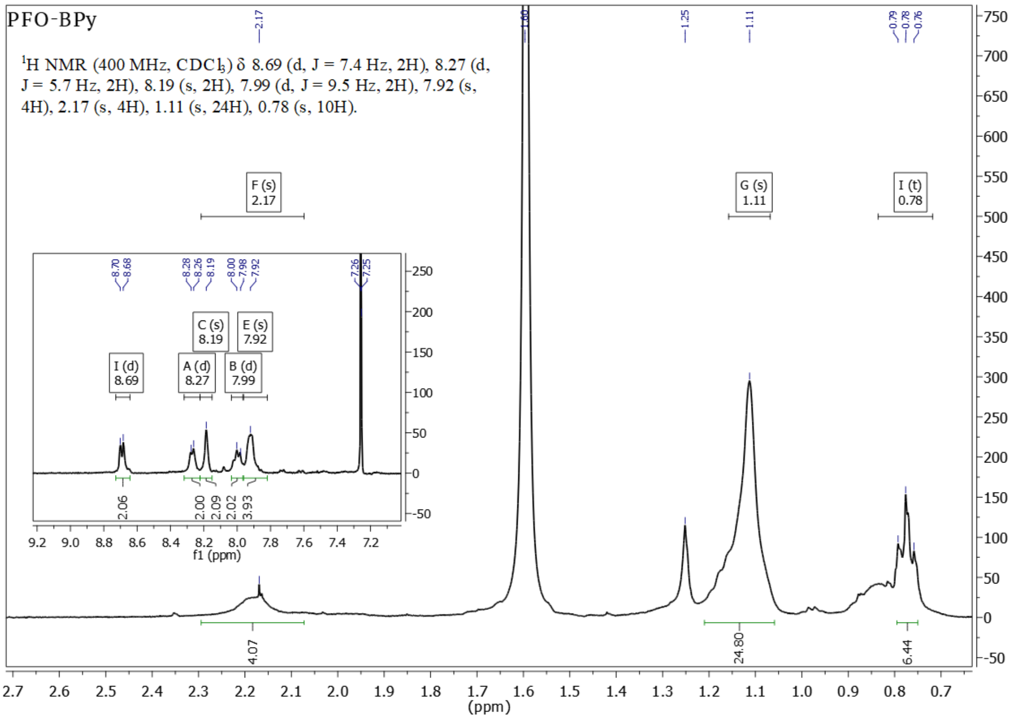

Figure S4 ^1H NMR spectrum of PFO-BPy6,6' synthesized in-house.

4.2. Deconvolution of optical absorbance spectra

The registered optical absorbance spectra were processed using the PTF Fit application as previously described. The resolution of peaks corresponding to individual chiralities, as illustrated in the examples presented in Figure S5, enabled estimation of SWCNT suspension composition and concentration of specific SWCNT species.

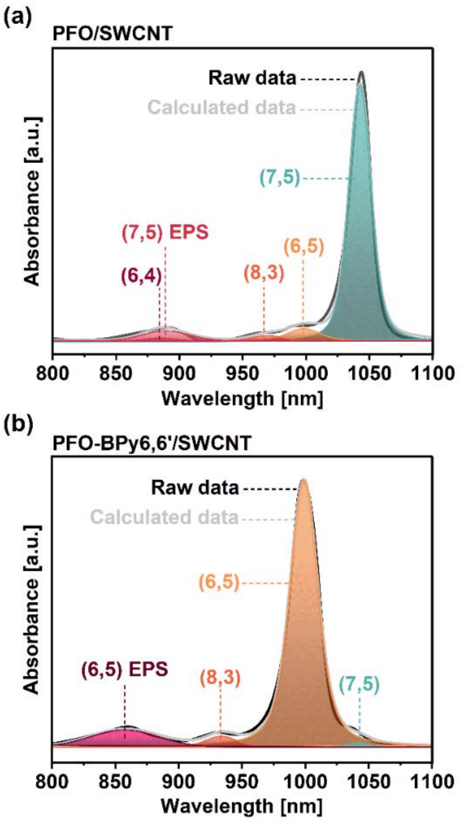

Figure S5 Deconvolution of optical absorbance spectra of SWCNTs suspended with (a) PFO and (b) PFO-BPy6,6'.

4.3. Enrichment factors for single-step CPE process

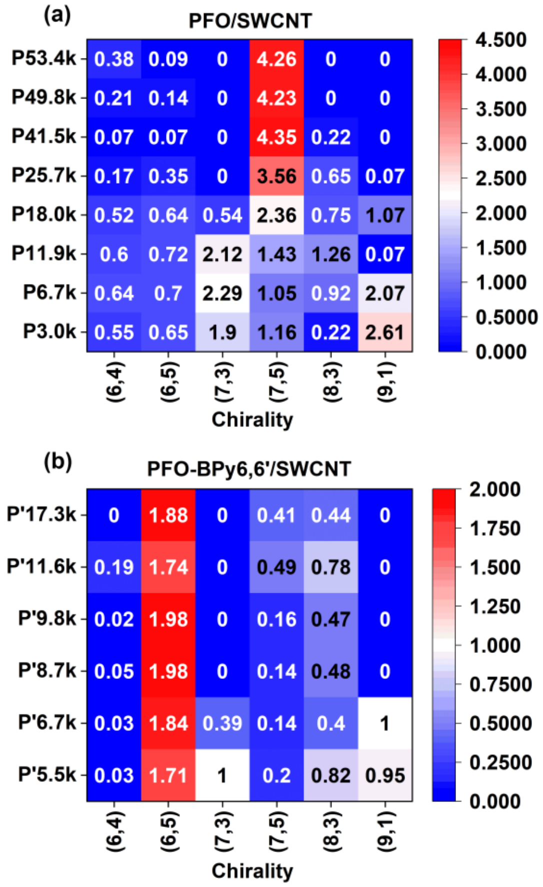

Figure S6 The enrichment factors were calculated for selected (a) PFO and (b) PFO-BPy6,6' polymers employed in the classical one-stage CPE process using deconvoluted optical absorbance spectra and the methodology explained above.

4.4. Determination of the structure of polymer coating around the isolated SWCNTs

To the best of our knowledge, the presence of multilayer polymer structure around SWCNTs has not yet been confirmed or disproved using experimental methods. This is a very challenging task since the characterization should be done in solution to find out, excluding the application of high-resolution electron microscopy. Moreover, theoretical work also does not sufficiently clarify this aspect since time- and resource-intensive modeling is usually engaged to study the deposition of single polymer molecules on the surface of SWCNTs in an idealized way.

In theory, AFM is a promising method that can be used to probe this phenomenon. Unfortunately, during sample preparation, the polymer's excess must be washed off. In the SWCNT dispersion, the amount of polymer is several times greater relative to the weight of dispersed SWCNTs, which would complicate or eliminate the possibility of sample visualization. Thus, it is likely that the polymer coating actually measured will differ from that produced by sonication and ultracentrifugation.

Nonetheless, we prepared (6,5) SWCNT/PFO-BPy6,6' and (7,5) SWCNT/PFO samples by CPE, subjected them to a mild purification treatment, and studied by AFM (Figure S7). The results showed that the polymer molecules formed multilayer structures around the SWCNTs, considering that the diameters of uncoated SWCNTs were only 0.757 nm and 0.829 nm, respectively, and the diameter values recorded by AFM were higher. The coating thickness was more significant in the case of PFO-suspended SWCNTs, which required a larger CP:SWCNT ratio to be effective. This polymer binds weaker to the SWCNT, compared to PFO-BPy6,6', according to our experience, so a larger weight excess needs to be applied so that the (7,5) SWCNTs suspended with PFO better resist ultracentrifugation. The provided images prove the existence of these multilayer structures and strongly suggest that their formation on the surface of SWCNTs is essential for the CPE.

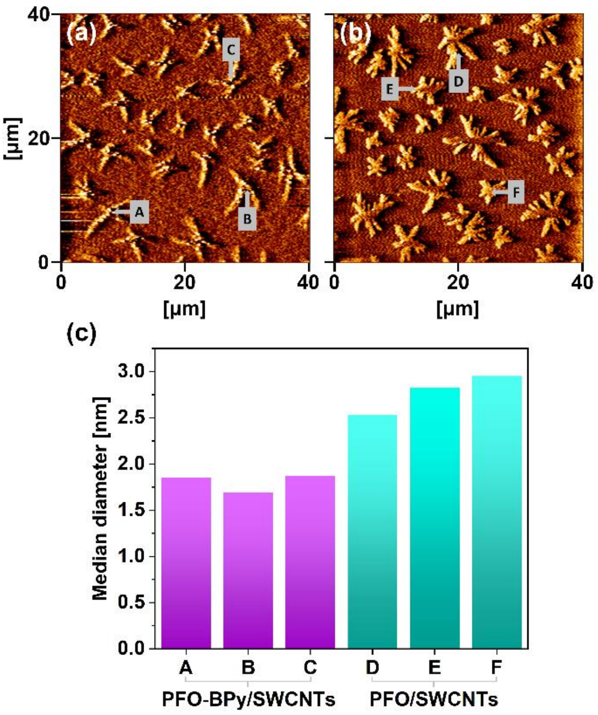

Figure S7 AFM images of SWCNTs sorted with (a) PFO-BPy (CP:SWCNT = 4:1) and (b) PFO (CP:SWCNT = 6:1). (c) Determination of the median diameter of these polymer/SWCNT multi-layer structures in three random locations of the samples.

4.5. Composition and concentration of SWCNT suspensions generated using multi-step CPE process

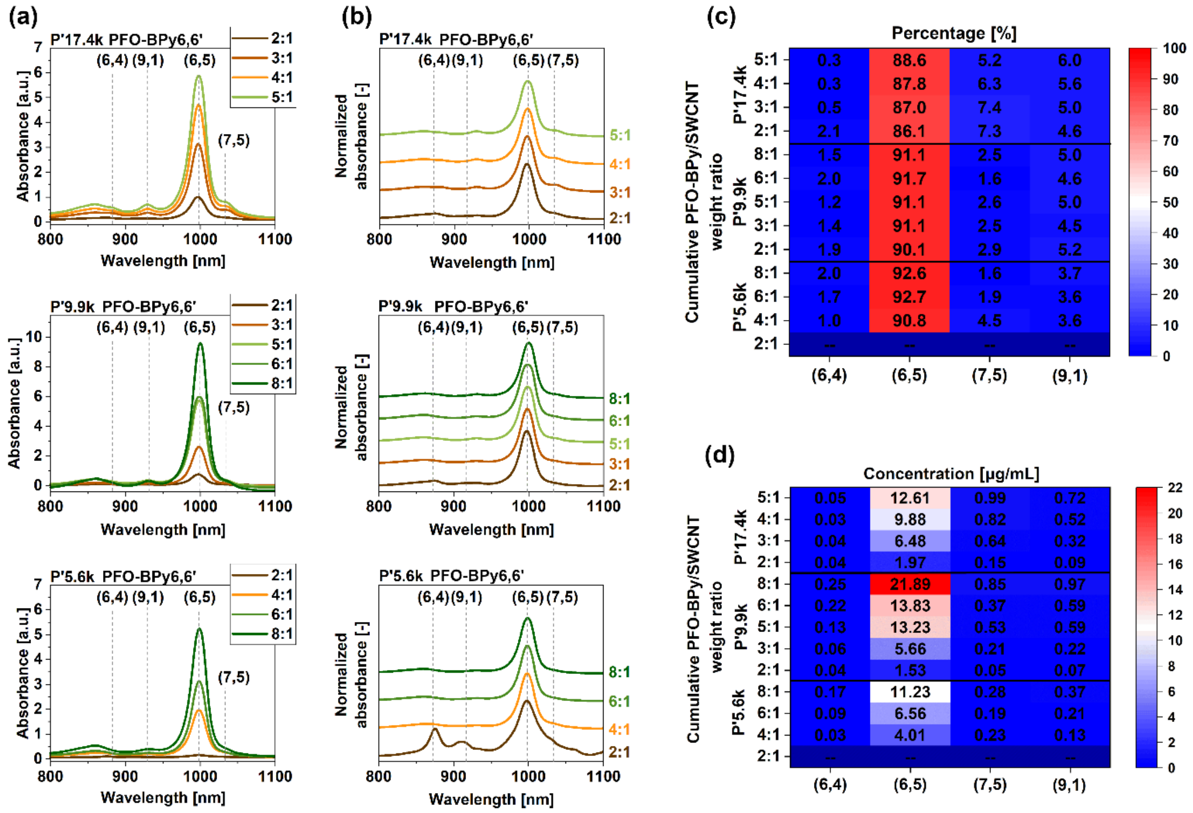

Figure S8 (a) Raw and (b) normalized optical absorbance spectra of SWCNTs suspended with P'5.6k, P'9.9k, and P'17.4k PFO-BPy6,6' batches at various CP:SWCNT ratios. (c) Percentage and (d) concentrations of individual SWCNT species obtained by various PFO-BPy6,6' batches in a multistep procedure quantified by deconvolution of corresponding optical absorbance spectra.

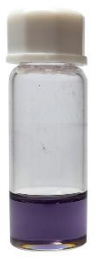

Figure S9 Photograph of SWCNTs purified using P'5.6k of PFO-BPy6,6' polymer at cumulative 8:1 ratio of polymer to SWCNTs.

4.6. Photoluminescence excitation-emission mapping of SWCNTs solubilized using polymers synthesized in this study

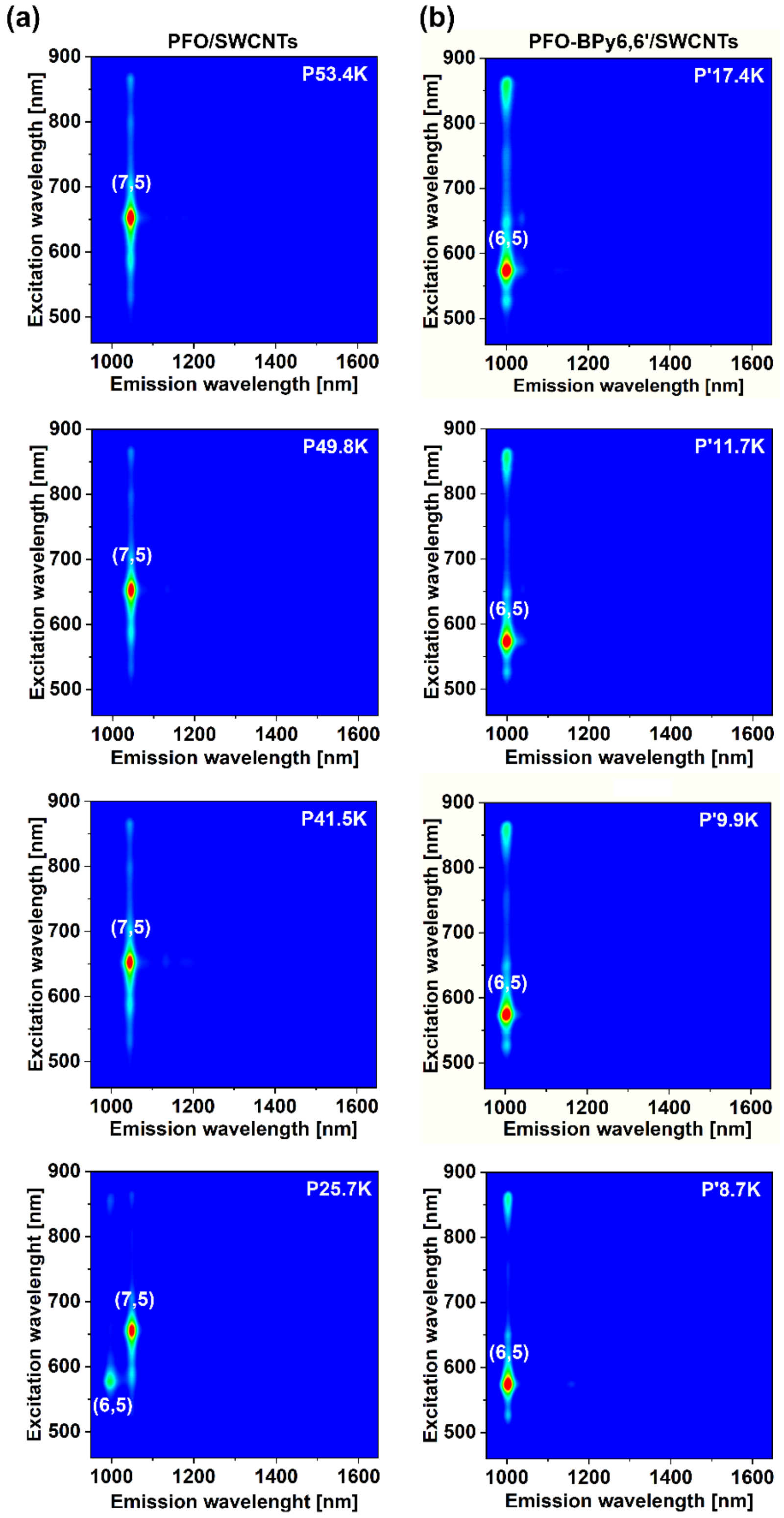

Figure S10 PL excitation-emission maps of SWCNT suspensions prepared with various indicated batches of (a) PFO and (b) PFO-BPy6,6.

4.7. Modeling of SWCNT-polymer interactions

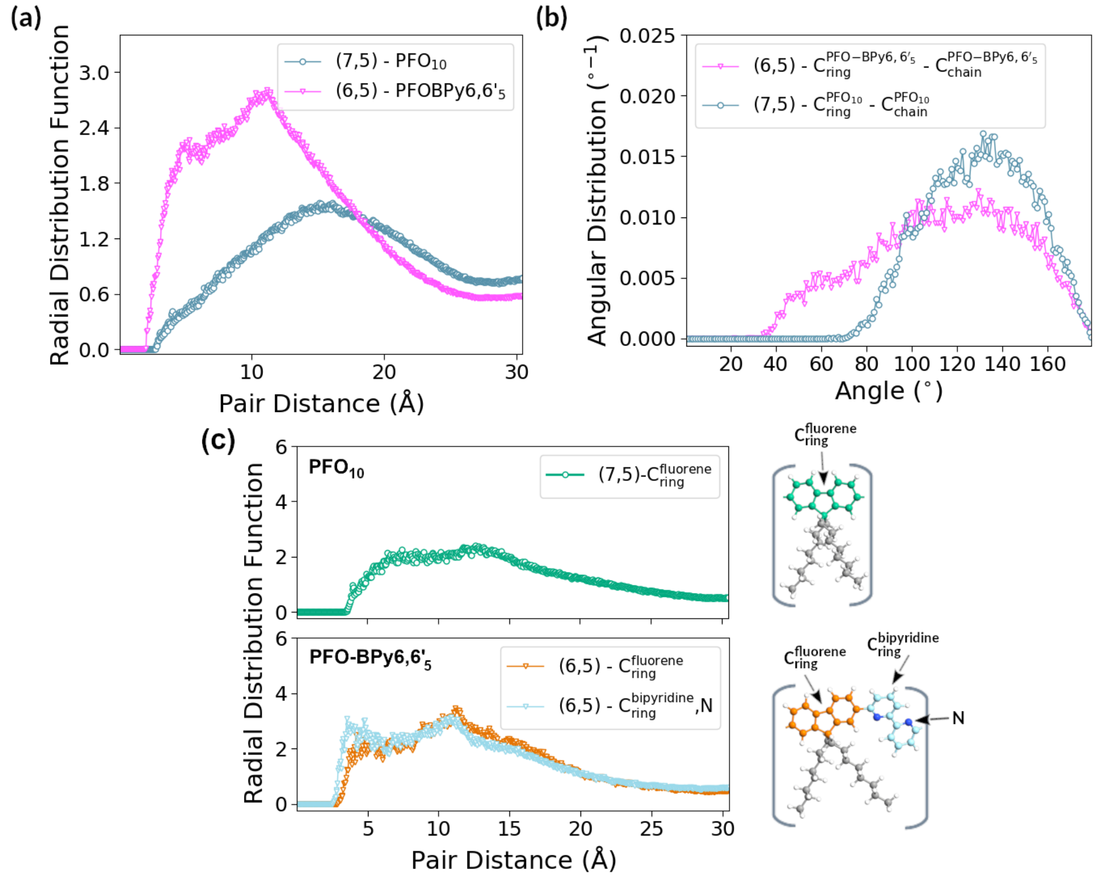

Figure S11 (a) Radial distribution functions (RDFs) between (6,5) SWCNT and PFO-BPy6,6' polymer and between (7,5) SWCNT and PFO polymer. (b) Angular distribution functions (ADFs) between SWCNT surface, polymer main chain carbon, and polymer side chain carbon atoms for both considered systems. (c) RDFs between (7,5) SWCNT and fluorene carbon atoms, between (6,5) SWNCT and fluorene carbon atoms, and between (6,5) SWCNT and bipyridine carbon atoms. Schematic diagrams showing fluorene and bipyridine units are displayed next to the RDFs. All carbon atoms constituting the polymer fluorene ring part are marked in teal in PFO polymer and in orange in PFO-BPy6,6' polymer. Carbon atoms forming bipyridine ring are light blue. Hydrogen and nitrogen atoms are marked in white and blue, respectively.

The RDF plot clearly showed that the probability of finding PFO-BPy6,6' polymer in the direct vicinity of (6,5) SWCNT was much higher than the probability of finding PFO polymer next to (7,5) SWCNT (cf. magnitude of RDF peaks shown in Figure S10a). It also strongly suggested that PFO-BPy6,6' was positioned much closer to (6,5) SWCNT than PFO to (7,5) (cf. positions of RDF peaks shown in Figure S10a). Further analysis indicated that both polymers were differently oriented with respect to SWCNTs. PFO-BPy6,6' maximized its wrapping around (6,5) SWCNT with both main and side chains, adjusting their positions to the lateral surface

of SWCNT. On the other hand, PFO polymer arranged only its main chain next to the SWCNT minimizing side chain contact with the SWCNT (cf. angle ranges in ADF plots shown in Figure S10b).

Analysis of the interaction between the fluorene unit, which is present in both polymers, with (6,5) and (7,5) SWCNTs revealed further differences in dissimilar behavior of PFO and PFO-BPy6,6′ polymers. As presented in Figure S10c, both (6,5)-$C_{ring}^{fluorene}$ and (7,5)-$C_{ring}^{fluorene}$RDFs produced two peaks. The first peak of the (6,5)-$C_{ring}^{fluorene}$ RDF appeared closer (at 4.7 Å) than the first peak of the (7,5)-$C_{ring}^{fluorene}$ RDF (at 7.4 Å), however, the magnitude of the second peak of the (6,5)-$C_{ring}^{fluorene}$RDF was visibly higher (3.5 Å) than the magnitude of the first peak of the (6,5)-$C_{ring}^{fluorene}$RDF (2.4 Å). This suggested that the fluorene unit would rather position itself further away from (6,5) SWCNT (at 11.2 Å). In the case of (7,5) SWCNT, the differences between magnitudes of the (7,5)-$C_{ring}^{fluorene}$ RDF peaks were much smaller. The first peak of the (7,5)-$C_{ring}^{fluorene}$ RDF had a value of 2.3 Å, while the second one had a value of 2.4 Å, indicating that it was almost equally probable to find a fluorene unit at 7.4 Å and at 12.6 Å from the lateral surface of (7,5) SWCNT.

Since the main chain of PFO-BPy6,6′ polymer was positioned considerably close to the lateral surface of (6,5) SWCNT (see Figure 5c) it must have been the bipyridine ring unit of PFO-BPy6,6′ polymer that brought C_{ring} close to the (6,5) SWCNT. Comparison between the (6,5)-$C_{ring}^{fluorene}$and (6,5)-$C_{ring}^{bipyridine}$RDFs indeed showed that the bipyridine ring unit preferred to position itself closer to the lateral surface of (6,5) SWCNT. The first peak of the (6,5)-$C_{ring}^{bipyridine}$appeared at 3 Å. The stronger interaction between the bipyridine unit and (6,5) SWCNT with respect to the interactions between the fluorene unit and (6,5) SWCNT may be attributed to the presence of nitrogen atoms in bipyridine unit and their absence in the fluorene unit of PFO-BPy6,6′ polymer.

4.8. The impact of the polymer composition on the capacity for sorting SWCNTs

Polymers are combinations of chains made up of different numbers of monomers. In order to describe the composition of such a non-homogeneous collective, scientists have defined specific quantitative parameters. The most commonly used parameters are the number-average molecular weight M_n, the weight-average molecular weight M_w, and the dispersity, which is their ratio $Đ = M_w / M_n$. For polymers obtained using controlled methods, e.g., anionic polymerization, it is possible to obtain polymers with an almost uniform chain length distribution, giving values of $Đ = 1 - 1.1$. Unfortunately, for polymers obtained using polycondensation methods, these values are usually between 1.5 and 6. This results in both very short and very long chains being present in a given polymer sample. In addition to the average molecular weight values for a given polymer, it is also useful to consider the cumulative molecular weight distribution, which informs us about the molecular weight for a given percentage of the studied polymer.

Regarding the use of conjugated polymers and oligomers for SWCNT sorting, based on the research work of scientists who synthesized oligomers of different lengths, i.e., from dimers to PDDF octamers, we know that both the sorting selectivity and the SWCNT dispersion efficiency are affected by molecular weight. Above a certain chain length, the selectivity between oligomers shows a much lower variation than the stability of SWCNT/polymer complexes. In essence, longer oligomers bind much stronger to SWCNTs, thereby improving their solubility in the liquid medium [41].

In light of the foregoing, we can discuss how the selectivity and isolation efficiency change for polymers with different M_w values. To support our reasoning, we provide cumulative molecular weight distributions for 5 polymer batches used in this study (Figure S12, Table S2). For PFO-BPy of moderate molecular weight of M_w = 9 kg/mol, about 10% of the polymer chains are unlikely to show any activity in CPE. At an AB monomer molecular weight of ca. 543 g/mol, the molecular weight of about 2 kg/mol corresponds to an ABAB molecule. On the other hand, PFO-BPy batches with large M_w values, such as 100 kg/mol and 118 kg/mol, are also inactive under the standard CPE process conditions presented in the publication. This is due to the fact that only about 10% of the polymer by weight is characterized by molecular weights below 25 kg/mol, which seems necessary to dissolve the material in toluene at room temperature.

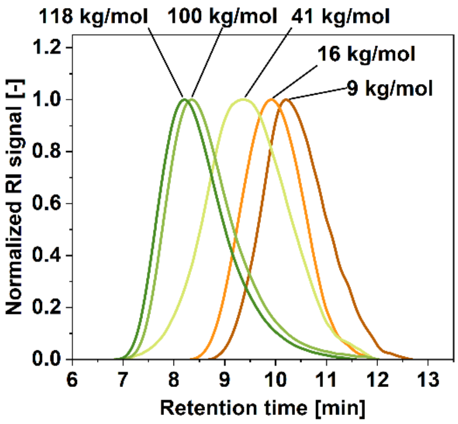

Figure S12 GPC chromatograms of the selected PFO-BPy6,6′ polymers obtained in this work.

Table S2 Molecular characteristics of the five selected PFO-BPy6,6′ batches of various polymer lengths.

10% of cumulative molecular weight [kg/mol]	Weight average molecular weight M_w [kg/mol]	90% of cumulative molecular weight [kg/mol]
2.1	9.0	18.9
3.4	15.7	34.2
5.7	41.2	94.2
19.1	98.5	192.1
25.9	118.1	226.5

For polymers with low M_w, the solubility problem of PFO-BPy6,6', which affects polymer batches with higher M_w, is marginalized. However, polymers of small M_w do not provide sufficient stability to the PFO-BPy/SWCNT complexes, which leads to the sedimentation of a considerable amount of them during centrifugation. Therefore, to alleviate this issue, one might increase the content of such polymers in the system. Consequently, the surface area of interaction between the polymer molecules and the SWCNTs is greatly increased, and the aforementioned goal is achieved. At the same time, PFO-BPy batches of small molecular weights contain only a small amount of heavy molecular-weight fractions, so when their concentration is increased, the polymer does not suffer solubility issues.

4.9. The impact of the processing conditions on the SWCNT sorting performance

To investigate whether the limited molecular mobility of the polymer chains could explain a lower-than-expected CPE performance, we studied the impact of the sonication time, power, and process mixture temperature (Figure S13). Experiments were performed for both PFO and PFO-BPy6,6' of moderate or high molecular weights, which should be prone to such limitations. The results proved that the initially chosen 8-minute sonication was an optimum time to reach a satisfactory degree of SWCNT solubilization in the liquid medium while simultaneously marginalizing the risk of SWCNT shortening due to cavitation or reducing the possibility of structural defect formation (Figure S13ab). Extension of the homogenization to 32 minutes offered a considerable benefit in terms of the amount of harvested monochiral (7,5) SWCNTs by PFO, judging by the notably increased absorbance value (Figure S13a). We concluded that within the short time of sonication (8 minutes), the system was limited by the kinetics of the PFO dissolution and deposition on the SWCNTs, especially since the employed batch had a relatively high molecular weight. On the other hand, the PFO-BPy6,6’/SWCNT system exhibited no such improvement when the sonication time was prolonged, strongly suggesting that the kinetic regime was not valid in this case (Figure S13b). The performance of PFO-BPy6,6’ was boosted when the sonication power was increased, indicating that higher energy had to be delivered to the system to overcome the thermodynamic limitations, leading to the self-aggregation of the polymer. These results once again prove the dissimilar nature of these two polymers.

Furthermore, we also evaluated PFO-BPy6,6’, which had a notably high molecular weight (M_w = 98.5 kg/mol) and should suffer the most from limited molecular mobility. The results matched the expectations, proving that the aforementioned constraints were in force. The typical homogenization conditions over an ice bath yielded almost no suspended SWCNTs (Figure S13c). Besides that, the polymer exhibited low solubility in toluene. However, when the temperature of the process mixture was increased, the solubility issue was alleviated, and an evident increase in SWCNT concentration was observed. In this case, the extension of the sonication time was also beneficial as it improved the molecular dynamics (Figure S13d).

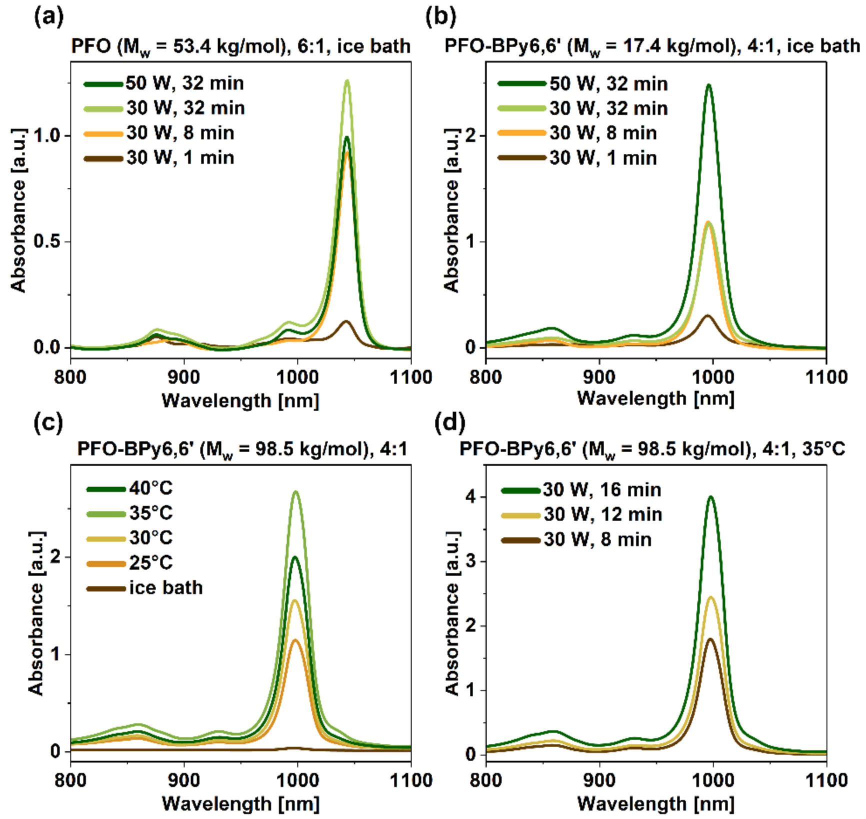

Figure S13 Optical absorbance spectra of SWCNTs suspended with (a) PFO (M_w = 53.4 kg/mol), (b) PFO-BPy6,6' (M_W = 17.4 kg/mol), (c,d) PFO-BPy6,6' (M_W = 98.5 kg/mol) under the specified processing conditions.

It has to be stressed that regardless of the employed processing conditions (8 min sonication at 30 W or 32 min sonication at 50 W), the degree of disorder in the material stayed at a notably low level. The D/G+ ratios measured by Raman spectroscopy were particularly low, i.e., equal to 0.02 (Figure S14a).

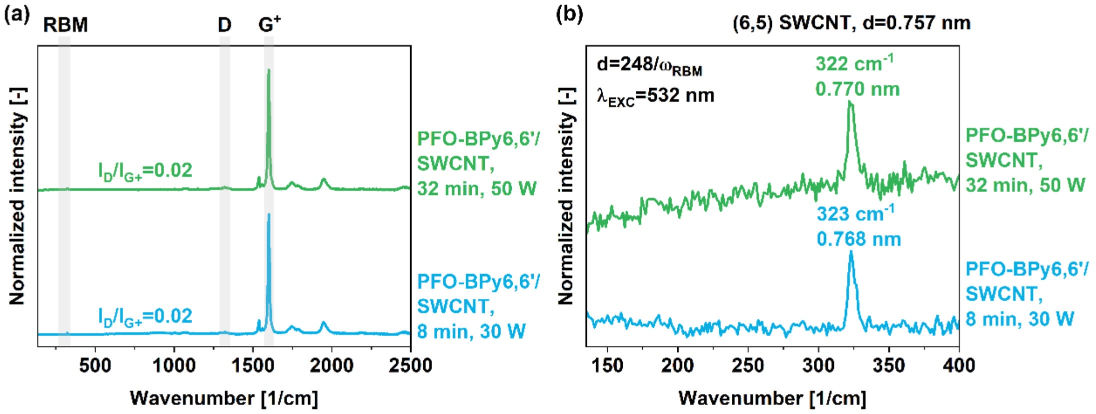

Figure S14 Raman spectra of SWCNTs sorted with PFO-BPy6,6' under different processing conditions. (a) full spectra, (b) magnification of the RBM area.

Most importantly, the created SWCNT suspensions were entirely composed of the desired semiconducting SWCNTs. No signs of metallic SWCNTs were detected while studying the radial breathing mode (RBM) area of the recorded spectra (Figure S14b).

4.10. Comparison of single- and multi-step CPE

To better illustrate the merits of the developed strategy, we conducted additional experiments using one of the challenging PFO batches, which exhibited decreased affinity to (7,5) SWCNTs (Figure S15). The total ratio of PFO to SWCNTs was 6:1 in each case. The first sample produced after 8 minutes of sonication produced a diluted SWCNT suspension containing a substantial amount of (6,5) SWCNT contamination. Then, we noted that using the same batch of PFO and employing a single 24-minute sonication improved both the yield and selectivity of the process since the amount of harvested (7,5) SWCNTs tripled. However, when a multi-step CPE process was also engaged for 24 minutes (3 x 8 minutes), in which the PFO:SWCNT ratio was gradually increased through 2:1 -> 4:1 -> 6:1, we witnessed a dramatic increase in the concentration of (7,5) SWCNTs.

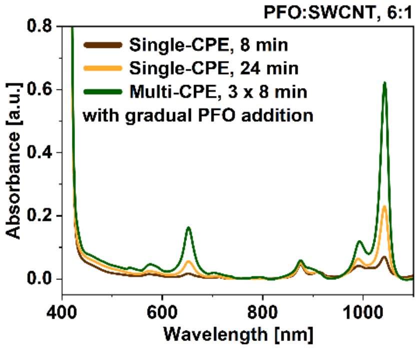

Figure S15 Optical absorbance spectra of PFO/SWCNT suspensions prepared using the specified conditions.

A stepwise increase in polymer concentration decreases its tendency to self-aggregate before it is adsorbed on the surface of SWCNTs. Consequently, the extraction system utilizes the polymer molecules more effectively. Concomitantly, the prolonged sonication and three centrifugation cycles (instead of one for a single-step CPE) facilitate reaching a proper alignment of the polymer molecules on the surface of SWCNTs. The polymer is subjected to more adsorption/desorption cycles during sonication, and the unstable PFO/SWCNT hybrids can be uncoupled during centrifugation.

4.11. Solubility issues of PFO-BPy6,6' affecting the extraction performance at high CP:SWCNT ratios

In contrast to PFO, PFO-BPy6,6' experienced solubility issues (Figure 4, Figure S7). When PFO-BPy6,6' (P'17.4k) was used in toluene at low to moderate concentrations (CP:SWCNT ratios ≤ 4:1, equivalent to 1.33 mg of polymer per mL of solvent), it was readily soluble. However, when the ratio was increased to 6:1 (2 mg/mL concentration), the amount of extracted SWCNTs did not increase proportionally.

To confirm that the lack of the expected performance increase was directly related to the problems with solubility of PFO-BPy, we pushed the synthetic limits of the obtained PFO-BPy6,6' up to M_w = 95.0 kg/mol. Then, we characterized the solubility of this polymer batch along with PFO-BPy6,6' (M_w = 17.4 kg/mol) and PFO (M_w = 81.7 kg/mol). The photographic evidence confirmed differences in the solubility of the two polymers (Figure S16).

PFO-BPy6,6' of large molecular weight was insoluble in toluene. Chlorobenzene had to be employed to solubilize it. In parallel, the same amount of PFO-BPy6,6' of intermediate molecular weight was found readily soluble in toluene. Interestingly, for PFO, it was possible to reach a very high concentration of this polymer in toluene without encountering any solubility issues, even though its weight-average molecular weight was much higher.

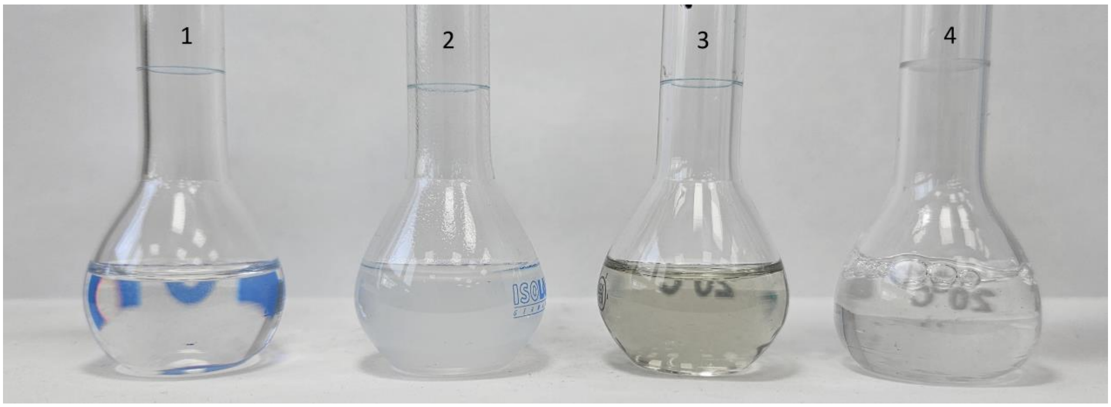

Figure S16 Photographs of polymer solutions: (1) 6.5 mg of PFO-BPy6,6' (M_w = 98.5 kg/mol) in 5 mL of chlorobenzene (concentration of 1.3 mg/mL) – the polymer was fully soluble, (2) 6.5 mg of PFO-BPy6,6' (M_w = 98.5 kg/mol) in 5 mL of toluene (concentration of 1.3 mg/mL) – the polymer was mostly insoluble, (3) 6.5 mg of PFO-BPy6,6' (M_w = 17.4 kg/mol) in 5 mL of toluene (concentration of 1.3 mg/mL) – the polymer was fully soluble, (4) 25 mg of PFO (M_w = 81.7 kg/mol) in 5 mL of toluene (concentration of 5 mg/mL) – the polymer was fully soluble.

5. Literature

[1] T. Yokozawa, A. Yokoyama, *Chem Rev* **2009**, *109*, 5595.

[2] T. Yokozawa, Y. Ohta, *Chem Rev* **2016**, *116*, 1950.

[3] J. Lee, H. Kim, H. Park, T. Kim, S.-H. Hwang, D. Seo, T. D. Chung, T.-L. Choi, *J Am Chem Soc* **2021**, *143*, 11180.

[4] H. Kim, J. Lee, T. Kim, M. Cho, T. L. Choi, *Angewandte Chemie - International Edition* **2022**, *61*, e202205828.

[5] A. Omrani, L. C. Simon, A. A. Rostami, M. Ghaemy, *Thermochim Acta* **2008**, *468*, 39.

[6] L. Metzler, T. Reichenbach, O. Brügner, H. Komber, F. Lombeck, S. Müllers, R. Hanselmann, H. Hillebrecht, M. Walter, M. Sommer, *Polym Chem* **2015**, *6*, 3694.

[7] T. Wasiak, D. Just, A. Dzienia, D. Łukowiec, S. Wacławek, A. Mielańczyk, S. Kodan, A. Bansal, R. Chandra, D. Janas, *Sci Rep* **2024**, *14*, 2336.

[8] P. Taborowska, A. Mielańczyk, A. Dzienia, D. Janas, *Submitted to Carbon* **2024**.

[9] W. Yi, A. Malkovskiy, Q. Chu, A. P. Sokolov, M. L. Colon, M. Meador, Y. Pang, *J Phys Chem B* **2008**, *112*, 12263.

[10] P. Hohenberg, W. Kohn, *Physical Review* **1964**, *136*, B864.

[11] W. Kohn, L. J. Sham, *Physical Review* **1965**, *140*, A1133.

[12] A. D. Becke, *J Chem Phys* **1993**, *98*, 5648.

[13] C. Lee, W. Yang, R. G. Parr, *Phys Rev B* **1988**, *37*, 785.

[14] S. H. Vosko, L. Wilk, M. Nusair, *Can J Phys* **1980**, *58*, 1200.

[15] P. J. Stephens, F. J. Devlin, C. F. Chabalowski, M. J. Frisch, *J Phys Chem* **1994**, *98*, 11623.

[16] S. Smidstrup, T. Markussen, P. Vancraeyveld, J. Wellendorff, J. Schneider, T. Gunst, B. Verstichel, D. Stradi, P. A. Khomyakov, U. G. Vej-Hansen, M.-E. Lee, S. T. Chill, F. Rasmussen, G. Penazzi, F. Corsetti, A. Ojanperä, K. Jensen, M. L. N. Palsgaard, U. Martinez, A. Blom, M. Brandbyge, K. Stokbro, *Journal of Physics: Condensed Matter* **2020**, *32*, 015901.

[17] https://www.synopsys.com/silicon/quantumatk.html, **2024**.

[18] H. Elhaes, M. Morsy, I. S. Yahia, M. Ibrahim, *Opt Quantum Electron* **2021**, *53*, 269.

[19] E. Chełmecka, K. Pasterny, T. Kupka, L. Stobiński, *J Mol Model* **2012**, *18*, 2241.

[20] M. Wykes, B. Milián-Medina, J. Gierschner, *Front Chem* **2013**, *1*, DOI 10.3389/fchem.2013.00035.

[21] G. Ye, W. Talsma, K. Tran, Y. Liu, S. Dijkstra, J. Cao, J. Chen, J. Qu, J. Song, M. A. Loi, R. C. Chiechi, *Macromolecules* **2022**, *55*, 1386.

[22] H. J. Monkhorst, J. D. Pack, *Phys Rev B* **1976**, *13*, 5188.

[23] E. C. Neyts, A. Bogaerts, *Theor Chem Acc* **2013**, *132*, 1320.

[24] K. M. Bal, E. C. Neyts, *J Chem Phys* **2014**, *141*, 204104.

[25] L. Martínez, R. Andrade, E. G. Birgin, J. M. Martínez, *J Comput Chem* **2009**, *30*, 2157.

[26] E. Bitzek, P. Koskinen, F. Gähler, M. Moseler, P. Gumbsch, *Phys Rev Lett* **2006**, *97*, 170201.

[27] H. J. C. Berendsen, J. P. M. Postma, W. F. van Gunsteren, A. DiNola, J. R. Haak, *J Chem Phys* **1984**, *81*, 3684.

[28] G. J. Martyna, M. L. Klein, M. Tuckerman, *J Chem Phys* **1992**, *97*, 2635.

[29] D. C. Liu, J. Nocedal, *Math Program* **1989**, *45*, 503.

[30] A. K. Rappé, C. J. Casewit, K. S. Colwell, W. A. Goddard, W. M. Skiff, *J Am Chem Soc* **1992**, *114*, 10024.

[31] J. Schneider, J. Hamaekers, S. T. Chill, S. Smidstrup, J. Bulin, R. Thesen, A. Blom, K. Stokbro, *Model Simul Mat Sci Eng* **2017**, *25*, 085007.

[32] U. Essmann, L. Perera, M. L. Berkowitz, T. Darden, H. Lee, L. G. Pedersen, *J Chem Phys* **1995**, *103*, 8577.

[33] A. K. Rappe, W. A. Goddard, *J Phys Chem* **1991**, *95*, 3358.

[34] J. E. Lennard-Jones, *Proceedings of the Royal Society of London. Series A, Containing Papers of a Mathematical and Physical Character* **1924**, *106*, 441.

[35] J. E. Lennard-Jones, *Proceedings of the Royal Society of London. Series A, Containing Papers of a Mathematical and Physical Character* **1924**, *106*, 463.

[36] J. E. Lennard-Jones, *Proceedings of the Physical Society* **1931**, *43*, 461.

[37] Y. Maeda, Y. Konno, A. Nishino, M. Yamada, S. Okudaira, Y. Miyauchi, K. Matsuda, J. Matsui, M. Mitsuishi, M. Suzuki, *Nanoscale* **2020**, *12*, 6263.

[38] X. Wei, T. Tanaka, S. Li, M. Tsuzuki, G. Wang, Z. Yao, L. Li, Y. Yomogida, A. Hirano, H. Liu, H. Kataura, *Nano Lett* **2020**, *20*, 410.

[39] M. Pfohl, D. D. Tune, A. Graf, J. Zaumseil, R. Krupke, B. S. Flavel, *ACS Omega* **2017**, *2*, 1163.

[40] N. Nair, M. L. Usrey, W.-J. Kim, R. D. Braatz, M. S. Strano, *Anal Chem* **2006**, *78*, 7689.

[41] N. Berton, F. Lemasson, F. Hennrich, M. M. Kappes, M. Mayor, *Chemical Communications* **2012**, *48*, 2516.
